# Geographical inequalities and temporal trends in pediatric cardiovascular diseases in Indonesia: a 34-year global burden of disease analysis

**DOI:** 10.3389/fpubh.2025.1688700

**Published:** 2025-12-16

**Authors:** Muhammad Iqhrammullah, Derren D. C. H. Rampengan, Ikhwan Amri, Roy Novri Ramadhan, Muhammad Haneef Ghifari, Firyal Khansa, Novi Kurnia Rizki, Starry H. Rampengan, Radityo Prakoso, Muhammad Habiburrahman

**Affiliations:** 1 Postgraduate Program of Public Health, Universitas Muhammadiyah Aceh, Banda Aceh, Indonesia; 2 Faculty of Medicine, Universitas Sam Ratulangi, Manado, Indonesia; 3 Department of Geography Education, Faculty of Teacher Training and Education, Universitas Samudra, Langsa, Indonesia; 4 Faculty of Medicine, Universitas Airlangga, Surabaya, Indonesia; 5 Medical Research Unit, School of Medicine, Universitas Syiah Kuala, Banda Aceh, Indonesia; 6 Department of Pharmacy, Faculty of Mathematics and Natural Sciences, Universitas Syiah Kuala, Banda Aceh, Indonesia; 7 Division of Interventional Cardiology, Department of Cardiology and Vascular Medicine, R.D. Kandou Central General Hospital, Universitas Sam Ratulangi, Manado, Indonesia; 8 Division of Pediatric Cardiology and Congenital Heart Disease, Department of Cardiology and Vascular Medicine, National Cardiovascular Center Harapan Kita, Universitas Indonesia, Jakarta, Indonesia; 9 Faculty of Medicine, Imperial College London, London, United Kingdom; 10 Faculty of Medicine, Universitas Indonesia, Jakarta, Indonesia

**Keywords:** cardiovascular disease, DALY, epidemiological trends, global burden of disease, health disparity

## Abstract

**Background:**

Pediatric cardiovascular diseases (CVDs), including congenital heart anomalies (CHAs), rheumatic heart disease (RHD), and related conditions, remain a significant health challenge in Indonesia, especially given the country’s diverse geography and disparities in healthcare access. We aim to analyze national and provincial trends in the burden of pediatric CVDs in Indonesia from 1990 to 2023 using Global Burden of Disease (GBD) data.

**Methods:**

We conducted a retrospective observational analysis using the GBD 2023 dataset, focusing on the prevalence, mortality, and disability-adjusted life years (DALYs) of pediatric cardiovascular diseases across age groups. Trends were assessed by sex and province. The Estimated Annual Percentage Change (EAPC) was calculated for each CVD subcategory and age group by fitting a log-linear regression model to the natural logarithm of annual rates. To evaluate nonlinear temporal patterns, generalized additive models (GAMs) were applied with penalized smoothing splines for year. Poisson or negative binomial regression models were used to model mortality counts, with the latter selected when overdispersion exceeded 1.5.

**Results:**

From 1990 to 2023, Indonesia showed an overall decline in DALY rates for CHAs, decreasing from 120,809.85 to 68,324.17 per 100,000, and for non-congenital CVDs, from 42,118.56 to 29,842.73 per 100,000. The most notable improvements occurred among infants and toddlers, whereas adolescents showed stagnant or rising burdens, particularly for ischemic heart disease (IHD), hypertensive heart disease (HHD), and aortic aneurysm. CHAs remained the leading contributor, with neonatal prevalence and mortality in 2023 reaching 1511.53 and 1341.68 per 100,000, respectively. Despite the overall national decline, positive EAPC values (*p* < 0.001) were observed for these adult-type cardiovascular conditions within the transitioning adolescent population. Regionally, the eastern provinces showed consistently higher mortality—CHA from 23.20 to 15.58 per 100,000 and non-congenital CVDs from 6.07 to 5.23 per 100,000.

**Conclusion:**

CHAs remain the leading cause of pediatric CVD in Indonesia, especially among neonates, while adolescents face rising adult-type cardiovascular conditions. National improvements are uneven, with eastern provinces experiencing higher burdens due to limited access to care. These inequalities highlight the need for targeted prevention, early detection, and strengthened long-term management to ensure equitable and sustainable child cardiovascular health.

## 1 Introduction

In 2021, cardiovascular diseases (CVDs) are the main cause for mortality and morbidity among Southeast Asia (SEA) countries, where Indonesia as the second top country with the highest Disability-Adjusted Life Year (DALY) counts (1). In a broader Asian region, the non-adjusted mortality attributable to CVDs was expected to experience 91.2% increase in 2025 (2). Interestingly, the mortality is expected to decline following the adjustment with age, which can be attributed to the trend in pediatric population (2). China, Japan, South Korea, India, and Singapore reported the decline in mortality attributable to CVDs up to 67.98% (3). The study further noted that the highest mortality was experienced by pediatric population aged less than 1 year (3). High burden of pediatric CVDs is often associated with low socioeconomic status (4, 5). Highly-dynamic epidemiology of pediatric CVDs pose a significant public health challenge, particularly in developing countries like Indonesia (6, 7).

Congenital heart anomalies (CHAs) and rheumatic heart disease (RHD) are among the most prevalent conditions, contributing substantially to childhood morbidity and mortality (6). Out of 4.2–4.8 million babies born annually, Indonesia may have 71,400 to 81,600 new CHA cases each year (8). Although advancements in pediatric cardiac surgery have been made, particularly since the establishment of the National Cardiovascular Center *Harapan Kita* Indonesia in 1985, disparities in healthcare access remain a pressing issue (9). Other than CHAs, pediatric heart failure was reported as significant burden this particular population, with 6.01 million cases was estimated globally (4). CHAs, cardiomyopathies, RHD, and chronic kidney disease (CKD) are attributable to the pediatric heart failure (4). Challenges such as limited medical personnel, uneven distribution of healthcare facilities, and a shortage of specialized training centers hinder timely interventions (10, 11). Understanding how the burden of pediatric CVDs has shifted over time is crucial for evaluating the effectiveness of past interventions and identifying emerging public health needs. Without this understanding, health programs risk being misaligned with current disease patterns and may fail to address regional disparities or shifting age-related risks.

A previous study has analyzed the trend in CVD burden in Indonesia, by using Global Burden of Disease (GBD) study data (12). They found that the mortality, morbidity, and prevalence rates are increasing in the country, which is in contrast to a declining trend in global or even SEA region (12). However, the previous study did not specifically examine the pediatric population, and therefore key age-specific and regional patterns in childhood CVD burden remain uncharacterized. Herein, the first comprehensive analysis focusing exclusively on the pediatric population is provided using GBD data from 1990 to 2023. It evaluates trends in prevalence, mortality, and DALYs for pediatric CVD in Indonesia, disaggregated by age groups (<28 days to 23 months and 2 to 19 years), sex, and region. The aim of this present study was to analyze temporal and geographic trends in the burden of pediatric CVDs in Indonesia from 1990 to 2023.

## 2 Methods

### 2.1 Study design

This retrospective observational study utilized data from the GBD Study 2023, provided by the Institute for Health Metrics and Evaluation (IHME), to analyze trends in the burden of pediatric CVDs from 1990 to 2023. The analysis focused on three key metrics: prevalence, mortality, and DALYs, with shifts over time examined across two pediatric age groups: <28 days, 1–5 months, 6–11 months, and 12–23 months, 2–4 years, 5–9 years, 10–14 years, and 15–19 years. The GBD study is a comprehensive and standardized source of global health data, providing estimates of disease burden across various population levels, including global, regional, national, and subnational data. It integrates data from vital registration systems, household surveys, disease registries, and other sources, ensuring consistent and comparable estimates.

Data on CHAs, CVDs (non-congenital), cardiomyopathy and myocarditis, endocarditis, hypertensive heart disease (HHD), ischemic heart disease (IHD), non-rheumatic valvular heart disease (VHD), pulmonary arterial hypertension (PAH), RHD, stroke, and other CVDs available for this age category were extracted using the Global Health Data Exchange (GHDx) query tool^1^. Variables analyzed included prevalence, mortality, and DALYs, stratified by sex and age group, for the years 1990 to 2023. The shifts (presented as percentage) in DALYs for each province were also collected. The data could picture the disease burden and trends, as suggested by previous reports (13, 14). In GBD 2023, all-cause mortality estimates were generated using OneMod, a unified model that replaced the previous mortality modeling framework based on model life tables and other systems, while DisMod-MR 2.1 continues to be used for estimating non-fatal disease outcomes (15, 16). OneMod directly estimates mortality across age, time, and location using both parametric (SpXMod) and non-parametric (KReg) components. Furthermore, the recent GBD is more precise for pediatric population due to the use of complete birth history data for <15 years. For this study, the focus was placed on examining the temporal shifts in DALYs within the specified age groups.

### 2.2 Data sources

The data used to estimate the burden of disease in Indonesia were derived using the ‘GBD Input Source Tools.’ A detailed list of input sources contributing to the GBD estimates for Indonesia was provided in a previous report (13). Age- and sex-specific population data were obtained from the Indonesia Population and Housing Census (1961–2010). Primary sources for cause-of-death data included the Basic Health Research surveys, the Cause of Death Survey, the Mortality Registration System Strengthening Project, and the Indonesia Sample Registration Systems. The GBD data input sources covered the period from 2011 to 2018. Additionally, data from significant national surveys, such as the Fifth National Health Service Survey and the Chronic Disease and Risk Factor Surveillance System conducted from 2013 to 2014, were incorporated into the GBD 2023 study. GBD 2023 supersedes all previous GBD rounds, using updated data and improved statistical models (15).

### 2.3 Disease and measurements

This study analyzed the burden of pediatric CVD using data from the GBD study, focusing on six major subcategories: CAHs, cardiomyopathy and myocarditis, CVDs (acquired), endocarditis, PAH, RHD, stroke, and other cardiovascular conditions. These diseases were selected based on the data availability. The analysis included estimates of prevalence, mortality, and DALYs. DALYs were calculated by summing years lived with disability (YLDs), obtained by multiplying disease prevalence by a disability weight specific to each condition, and years of life lost (YLLs) due to premature mortality, derived from the number of deaths and the expected remaining years of life based on a standard life table, as described previously (17).

In GBD 2023, mortality estimation incorporated five covariates known to influence all-cause mortality: the socio-demographic index (SDI), HIV mortality rate, COVID-19 mortality rate, an island indicator, and the aggregate population attributable fraction (PAF) for all risk factors combined, excluding deaths due to HIV/AIDS and stochastic events. Uncertainty in all estimates was propagated through 1,000 draw-level simulations for each location-year-sex combination, with 95% uncertainty intervals (UIs) determined from the 2.5th and 97.5th percentiles of these draws. Additionally, the GBD 2023 framework modeled expected mortality as a function of SDI using a meta-regression with Bayesian regularized trimmed (MR-BRT) approach.

In the 2023 estimates, GBD employed an updated suite of modeling tools to generate internally consistent estimates of disease burden (15, 16). The new OneMod framework was introduced for all-cause mortality estimation, integrating SpXMod, a stagewise generalized linear model accounting for age-specific covariate effects, and KReg, a non-parametric kernel regression model that smooths residuals across age, time, and location. These improvements replaced the use of multiple separate life table systems in earlier GBD iterations, enhancing the transparency and precision of mortality estimation. For cause-specific mortality, the Cause of Death Ensemble Model (CODEm) continued to integrate multiple data sources and covariates to assess trends across locations and time. Disease incidence, prevalence, and mortality estimates were generated using DisMod-MR 2.1, a Bayesian meta-regression tool that ensures internal consistency across epidemiological parameters. In addition, the Spatiotemporal Gaussian Process Regression (ST-GPR) framework was used to model covariates and adjust for spatial and temporal variations, thereby refining the estimates at subnational levels.

For the present report, the estimates were stratified by specific pediatric age groups (<28 days, 1–5 months, 6–11 months, and 12–23 months, 2–4 years, 5–9 years, 10–14 years, and 15–19 years), sex, and two key time points, 1990 and 2023, to assess trends over three decades. The GBD data aggregation process integrated input from global, regional, and country-specific sources. This meant that the estimates were not solely based on data from Indonesia, but also included modeled values derived from similar populations where local data was limited or unavailable. Further details of the GBD methodology, including model specifications and data sources, are available in the GBD reference publications (14).

### 2.4 Statistical analysis

The primary statistical method employed in this analysis was the calculation of the Estimated Annual Percentage Change (EAPC). This metric was used to evaluate temporal trends in pediatric cardiovascular disease metrics across all locations and for both sexes. The analysis aimed to determine the annual rate of change for each disease measure over the study period, providing a quantitative assessment of shifting trends. To compute the EAPC following the standard estimation (18), data for each combination of disease, metric, and location were log-transformed to linearize the temporal trends. A simple linear regression model, defined as ln(y) = *α* + *βx* + *ε*, was applied, where ‘*y*’ represents the disease rate, ‘*x*’ is the calendar year, ‘*a*’ and ‘*b*’ are regression coefficients, and ‘*e*’ is the error term. The parameter ‘β’ indicates the rate of change in the logarithmic scale over time. The EAPC was derived from the equation: EAPC = 100 * (exp(*β*) − 1), which estimates the average percentage change per year. Positive EAPC values indicate an increasing trend, while negative values reflect a decline (18, 19). Statistical significance for EAPC was determined at *p* < 0.05, corresponding to 95% confidence intervals that do not include zero. All statistical computations were performed using the R programming language operated on Rstudio version 2024.04.2.

Mortality and population data for individuals aged below 20 years in Indonesia were extracted from the GBD 2023 dataset. Annual mortality counts were modeled using generalized linear models with a log link function and the logarithm of population as an offset term to estimate mortality rates per 100,000 population. Poisson regression was initially applied, and overdispersion was evaluated by comparing the Pearson residual deviance to the degrees of freedom; when overdispersion exceeded 1.5, a Negative Binomial model was used. To assess regional variation, Indonesia was divided into western and eastern regions based on provincial classification, and separate models were fitted for each cause, including region and year as covariates to capture geographic and temporal effects. Predicted mortality rates with 95% confidence intervals were derived from the fitted models and plotted to visualize temporal and regional trends from 1990 to 2023. The models were constructed for two causes, namely CHAs and non-congenital CVDs.

### 2.5 Geospatial visualization

To visualize the spatial and temporal shifts in pediatric cardiovascular burden, geospatial mapping was conducted using Tableau Public 2023.3 (Tableau Software, LLC, Seattle, WA, USA). Provincial-level DALY estimates for CHAs and other cardiovascular diseases among the pediatric population aged <20 years were extracted and integrated with Indonesia’s administrative boundary shapefiles obtained from the Geospatial Information Agency (*Badan Informasi Geospasial*, BIG). Choropleth maps were generated to depict the shifting distribution of DALYs across provinces from 1990 to 2023, with color gradients representing relative burden intensity.

### 2.6 Sensitivity analysis

A sensitivity analysis was conducted to examine the consistency of temporal patterns in DALY rates between the GBD 2021 and GBD 2023 datasets. Data were extracted for individuals under 20 years of age in Indonesia, focusing on CHAs and non-congenital CVDs. Generalized additive models (GAMs) were fitted separately for each dataset using the *mgcv* package in R (version 2025.09.1). The models employed a smooth term for year [s(year, *k* = 10)] and were estimated using restricted maximum likelihood (REML). Model performance was evaluated using the effective degrees of freedom (edf), *F*-statistic, adjusted *R*^2^, and percentage of deviance explained. Comparative plots of fitted smooths with 95% confidence intervals were generated to visualize potential discrepancies between datasets.

## 3 Results

### 3.1 Epidemiology of CHAs and non-congenital CVDs

Burdens of CHAs and pediatric CVDs are presented in Tables 1, 2. The highest burden of pediatric CVDs was observed in the <28 days age group, with a DALY rate of 3,567.01 (95% UI: 2,313.91–5,171.52) per 100,000 live births. The mortality rate attributable to CVDs in this group reached 43.13 (95% UI: 25.13–69.39) per 100,000 live births in females and 36.33 (95% UI: 17.89–57.99) per 100,000 live births in males. In the 1–5-month-old group, both DALY and mortality rates declined by more than half, to 1,650.64 (95% UI: 976.90–2,528.28) and 18.35 (95% UI: 10.84–28.13) per 100,000 live births, respectively. A declining trend in mortality accompanied by an increase in prevalence was observed in the older pediatric age groups. DALY rates decreased to 130.42 (95% UI: 94.34–171.61) per 100,000 in the 5–9-year group, then rose again to 676.14 (95% UI: 492.22–888.69) per 100,000 in the 15–19-year group. In this older group, females exhibited higher DALY rates, reaching 827.41 (95% UI: 564.90–1,231.94) per 100,000.

**Table 1:** Non-congenital CVD burdens among pediatric population expressed in rate per 100,000.

Sex and age	Prevalence		Deaths		DALY	
	Value (95% IU)	*Δ* (%)	Value (95% IU)	*Δ* (%)	Value (95% IU)	Δ (%)
<28 days						
Both	6.21 (4.62–8.80)	16.34	39.64 (25.71–57.47)	−1.32	3567.01 (2313.91–5171.52)	71.3
Female	6.79 (4.99–9.86)	14.88	43.13 (25.13–69.39)	−18.7	3881.69 (2261.70–6244.15)	36.66
Male	5.66 (4.28–7.81)	13.8	36.33 (17.89–57.99)	−21.52	3269.41 (1610.29–5218.33)	15.56
1–5 months						
Both	33.28 (25.96–43.89)	9.51	18.35 (10.84–28.13)	−34.13	1650.64 (976.90–2528.28)	−20.67
Female	34.58 (26.79–45.02)	9.35	19.53 (10.12–33.08)	−35.32	1757.14 (912.30–2973.90)	−22.82
Male	32.04 (24.89–41.66)	8.91	17.23 (8.21–30.35)	−35.61	1549.79 (740.58–2727.50)	−26.66
6–11 months						
Both	100.35 (78.19–129.97)	0.06	4.58 (2.57–7.16)	−58.89	418.14 (240.31–649.01)	−59.18
Female	102.43 (78.69–132.28)	−0.37	5.24 (2.35–9.82)	−63.25	477.63 (218.74–885.42)	−64.07
Male	98.39 (75.96–131.40)	−0.73	3.96 (1.75–6.80)	−65.9	361.77 (163.72–616.17)	−80.88
12–23 months						
Both	121.34 (100.71–148.38)	10.49	1.62 (0.96–2.40)	−31.54	156.66 (98.05–226.00)	−11.83
Female	113.76 (94.67–137.77)	10.23	1.77 (0.89–3.09)	−31.77	170.40 (92.13–290.66)	−14.51
Male	128.53 (104.53–158.97)	9.99	1.48 (0.73–2.44)	−31.95	143.62 (74.93–228.30)	−17.55
2–4 years						
Both	192.24 (166.12–223.60)	7.21	1.10 (0.63–1.64)	−37.48	118.40 (80.33–166.98)	−39.68
Female	194.09 (167.35–223.36)	7.16	1.14 (0.57–1.92)	−42.75	124.04 (76.69–195.65)	−43.42
Male	190.48 (162.58–224.67)	5.94	1.06 (0.52–1.83)	−43.24	113.02 (65.25–182.18)	−47.14
5–9 years						
Both	400.16 (329.15–476.77)	3.56	1.05 (0.66–1.52)	−45.82	130.42 (94.34–171.61)	−51.7
Female	404.85 (334.77–485.05)	3.42	1.25 (0.63–2.01)	−48.54	151.77 (98.19–216.87)	−53.21
Male	395.66 (319.11–469.74)	1.31	0.85 (0.45–1.47)	−54.35	110.00 (73.09–160.88)	−55.62
10–14 years						
Both	619.70 (501.99–745.48)	12.22	2.29 (1.53–3.20)	−24	242.39 (176.59–316.60)	3.76
Female	601.02 (495.66–725.38)	11.99	2.53 (1.43–3.94)	−30.05	265.18 (178.21–377.78)	−2.75
Male	637.37 (506.09–788.31)	11.14	2.07 (1.18–3.32)	−30.89	220.85 (146.82–321.27)	−5.99
15–19 years						
Both	831.49 (716.33–966.86)	8.58	8.15 (5.70–11.08)	−35.66	676.14 (492.22–888.69)	−29.86
Female	839.14 (728.46–977.99)	8.14	10.13 (6.56–15.64)	−35.94	827.41 (564.90–1231.94)	−34.12
Male	824.33 (705.21–965.79)	7.86	6.29 (3.51–9.88)	−36.29	534.34 (336.69–786.33)	−36.57

**Table 2:** CHA burdens among pediatric population expressed in rate per 100,000.

Sex and age	Prevalence		Deaths		DALY	
	Value (95% IU)	Δ (%)	Value (95% IU)	Δ (%)	Value (95% IU)	Δ (%)
<28 days						
Both	1511.53 (1247.34–1806.59)	45.08	1341.68 (844.75–1989.00)	25.97	120809.85 (76113.69–179029.90)	91.72
Female	1453.99 (1204.54–1734.94)	8.96	1051.57 (495.75–1743.09)	6.42	94707.70 (44708.79–156908.45)	10.57
Male	1565.95 (1284.02–1868.41)	3.3	1616.05 (799.02–2750.68)	1.94	145496.05 (72002.88–247617.53)	4.79
1–5 months						
Both	1011.83 (844.54–1190.15)	−4.06	174.08 (112.76–249.08)	−4.35	15690.43 (10166.42–22398.23)	−4.33
Female	985.91 (821.19–1164.63)	−4.47	146.65 (68.96–247.96)	−4.89	13227.80 (6259.97–22318.33)	−4.6
Male	1036.37 (870.33–1220.20)	−4.98	200.06 (107.22–330.63)	−5.08	18022.62 (9692.12–29771.88)	−5.95
6–11 months						
Both	740.05 (618.22–877.99)	−43.79	48.93 (30.46–74.23)	−44.38	4425.80 (2779.83–6689.07)	−47.81
Female	731.08 (609.46–872.31)	−49.99	43.84 (20.90–74.04)	−52.85	3969.84 (1925.96–6672.39)	−54.8
Male	748.54 (622.67–889.59)	−57.69	53.76 (28.94–99.41)	−63.93	4857.78 (2639.59–8934.14)	−74.41
12–23 months						
Both	617.44 (519.26–725.39)	−0.74	10.38 (6.17–15.55)	−2.21	971.43 (624.15–1427.26)	−1.13
Female	608.92 (507.58–728.59)	−2.35	9.02 (4.19–15.12)	−3.05	849.02 (420.29–1389.05)	−2.91
Male	625.53 (526.92–729.71)	−3.81	11.66 (6.07–20.08)	−3.42	1087.59 (590.36–1845.12)	−3.96
2–4 years						
Both	417.31 (356.31–478.60)	−19.95	3.90 (2.44–5.74)	−20.99	376.87 (253.38–539.67)	−21.71
Female	400.72 (344.19–465.99)	−21.83	3.10 (1.52–5.15)	−22.56	305.00 (169.58–480.82)	−22.55
Male	433.11 (370.03–494.58)	−22.88	4.66 (2.57–8.06)	−24.37	445.30 (253.23–730.80)	−24.54
5–9 years						
Both	218.19 (190.67–251.60)	−28.98	1.57 (0.99–2.33)	−29.39	146.32 (97.04–210.35)	−30.9
Female	204.55 (180.21–232.80)	−31.72	1.33 (0.71–2.09)	−32.64	124.34 (74.36–188.60)	−34.81
Male	231.24 (199.98–271.43)	−38.41	1.80 (0.90–3.13)	−41.1	167.34 (92.82–277.18)	−43.28
10–14 years						
Both	164.65 (141.10–191.21)	1.18	1.73 (1.17–2.55)	1.08	144.80 (99.69–206.40)	1.83
Female	162.00 (140.39–185.84)	1.01	1.40 (0.81–2.19)	0.48	118.31 (73.17–178.68)	0.48
Male	167.16 (142.18–195.91)	−0.18	2.05 (1.16–3.30)	0.38	169.85 (98.69–268.65)	−0.53
15–19 years						
Both	151.42 (130.67–174.24)	−11.08	1.73 (1.16–2.52)	−5.95	134.20 (89.53–191.36)	−11.15
Female	152.45 (131.88–173.62)	−13.06	1.54 (0.83–2.48)	−13.65	120.17 (68.88–188.03)	−13.85
Male	150.45 (128.02–174.81)	−16.01	1.91 (1.08–3.07)	−15.16	147.35 (87.60–233.31)	−18.61

Within the CHA category, both DALY and mortality rates were markedly higher in early life, reaching 120,809.85 (95% UI: 76,113.69–179,029.90) and 1,341.68 (95% UI: 844.75–1,989.00) per 100,000 in the <28 days age group. These values declined sharply in the 1–5-month group, with mortality and DALY rates decreasing to 174.08 (95% UI: 112.76–249.08) and 15,690.43 (95% UI: 10,166.42–22,398.23) per 100,000, respectively. The downward trend continued through adolescence, reaching 1.73 (95% UI: 1.16–2.52) for mortality and 134.20 (95% UI: 89.53–191.36) for DALY in the 15–19-year group. In contrast to non-congenital CVDs, CHA prevalence exhibited a steady decline with age, from 1,511.53 (95% UI: 1,247.34–1,806.59) per 100,000 in the <28 days group to 151.42 (95% UI: 130.67–174.24) per 100,000 in the 15–19-year group. Furthermore, DALY rates for CHAs were lower than those for non-congenital CVD causes in the 10–14 and 15–19-year age groups.

### 3.2 Epidemiology of non-congenital CVD subtypes

Among the CVD subtypes, the highest prevalence rates in the 0–23-month age range were attributable to stroke—38.29 (95% UI: 33.05–43.54) per 100,000 in the 12–23-month group—and cardiomyopathy, 19.18 (95% UI: 13.55–26.16) per 100,000 in the same age group. RHD was observed only in the 12–23-month group, with a prevalence of 4.94 (95% UI: 3.12–7.16) per 100,000. The prevalence of cardiomyopathy peaked at 21.41 (95% UI: 14.94–29.34) per 100,000 in the 2–4-year group and 22.97 (95% UI: 14.23–34.20) per 100,000 in the 15–19-year group. Stroke prevalence increased sharply with age, reaching 80.01 (95% UI: 70.12–91.24) per 100,000 in the 2–4-year group and rising further through childhood and adolescence to 148.29 (95% UI: 126.18–173.31) in 5–9 years, 226.22 (95% UI: 191.93–261.52) in 10–14 years, and 316.07 (95% UI: 269.71–365.36) per 100,000 in 15–19 years. Similarly, RHD burden increased rapidly during adolescence, reaching 310.90 (95% UI: 209.01–464.63) per 100,000 in the 15–19-year group. IHD and HHD began to emerge in this older group, with prevalences of 96.33 (95% UI: 63.58–136.07) and 2.54 (95% UI: 1.48–4.25) per 100,000, respectively.

Not only its high prevalence, stroke also contributed substantially to mortality, with an overall rate of 28.11 (95% UI: 17.84–44.32) per 100,000, and a higher burden among females—31.61 (95% UI: 15.96–52.21) per 100,000. Unlike its prevalence trend, stroke mortality declined sharply with age, reaching 0.41 (95% UI: 0.23–0.66) per 100,000 in the 5–9-year group. The rate increased again during adolescence, rising to 0.71 (95% UI: 0.41–1.15) in 10–14 years and 2.86 (95% UI: 1.73–4.33) in 15–19 years. In the <28 days group, mortality due to PAH and cardiomyopathy and myocarditis was 2.91 (95% UI: 0.49–8.47) and 1.43 (95% UI: 0.51–9.70) per 100,000, respectively, both of which declined progressively with age. RHD mortality was 0.16 (95% UI: 0.07–0.31) per 100,000 in 12–23 months, increasing to 1.45 (95% UI: 0.79–2.45) per 100,000 in 15–19 years. Among older adolescents, IHD and HHD mortality reached 2.87 (95% UI: 1.77–4.26) and 0.10 (95% UI: 0.05–0.16) per 100,000, respectively.

Cardiomyopathy and myocarditis showed the highest DALY rates in the early neonatal period, recorded at 398.72 (95% UI: 135.58–872.55), 368.15 (95% UI: 142.14–708.37), and 139.83 (95% UI: 59.64–251.78) per 100,000 in the <28 days, 1–5 months, and 6–11 months groups, respectively, with a steady decline thereafter through the 10–14-year group. Similarly, endocarditis exhibited a declining pattern, with DALY rates of 67.84 (95% UI: 26.87–158.20), 73.02 (95% UI: 27.96–146.89), and 24.79 (95% UI: 9.63–48.10) per 100,000 across the same age groups. PAH followed a comparable trend, showing DALY rates of 261.55 (95% UI: 43.72–761.83), 54.17 (95% UI: 11.40–152.44), and 18.87 (95% UI: 4.68–59.53) per 100,000 in the <28 days, 1–5 months, and 6–11 months groups, respectively, before increasing slightly in the 15–19-year population.

Stroke demonstrated a distinct trajectory, with DALYs of 2,529.97 (95% UI: 1,605.91–3,987.94) per 100,000 in <28 days, followed by a decline in infancy and a subsequent rise beginning in the 2–4-year group, continuing steadily to 265.64 (95% UI: 180.36–380.33) per 100,000 in 15–19 years. RHD displayed a progressive increase, first appearing at 14.19 (95% UI: 6.26–27.71) per 100,000 in 12–23 months, and rising to 121.48 (95% UI: 70.61–195.66) per 100,000 in 15–19 years. IHD contributed notably to the burden in late adolescence, with a DALY rate of 212.30 (95% UI: 132.18–312.35) per 100,000 in 15–19 years, higher among females at 270.20 (95% UI: 146.03–446.40) per 100,000. The summaries of prevalence, mortality and DALY rates of CVD subtype stratified by age are presented in Supplementary Tables S1–S6.

### 3.3 Shifting burdens in CHAs and non-congenital CVDs

**Figure 1:**
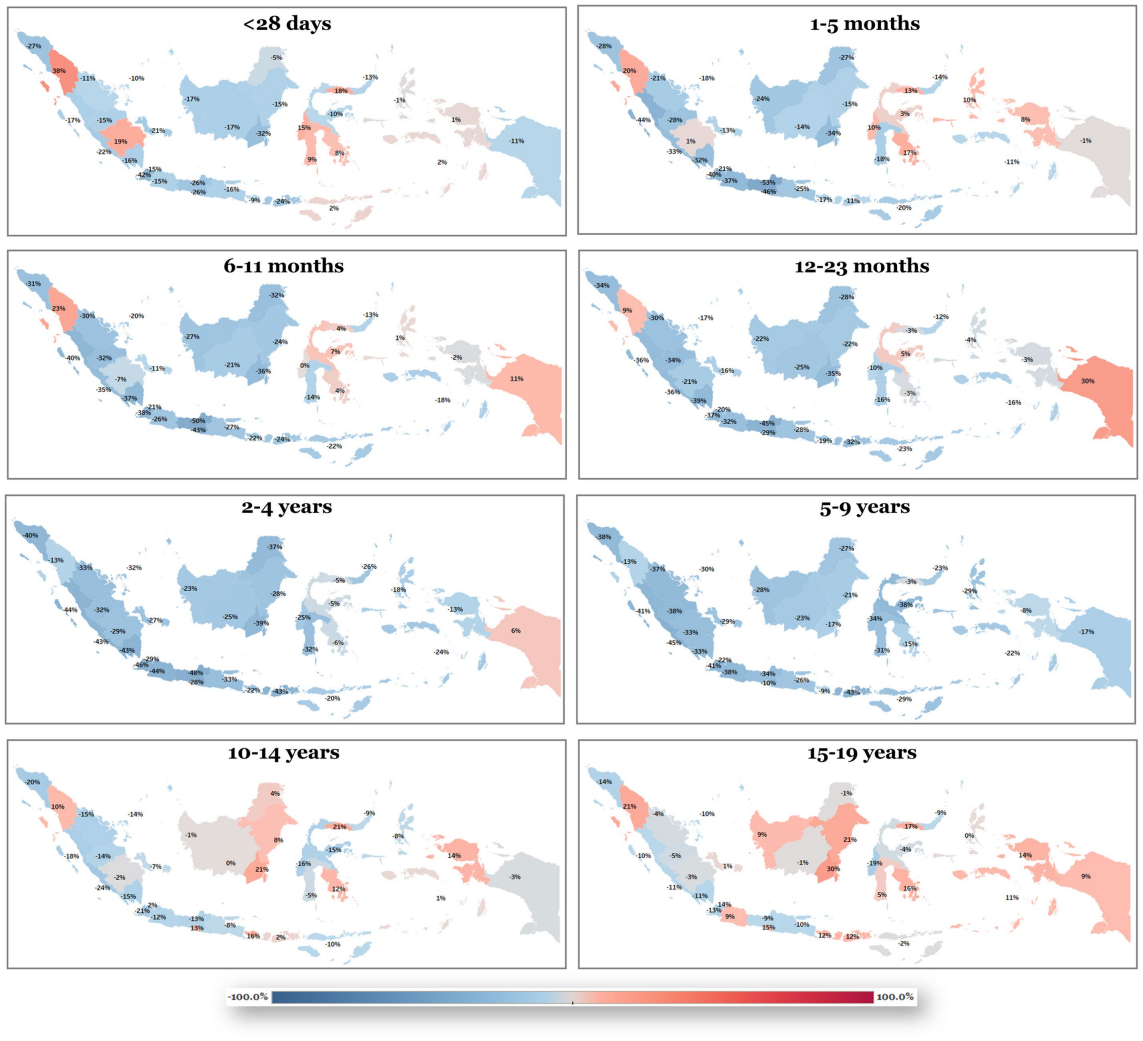
Shift in disability-adjusted life-years (DALYs) for congenital heart anomalies (CHAs) from 1990 to 2023 across pediatric age groups. Blue shading indicates improvement, and red shading indicates worsening.

**Figure 2:**
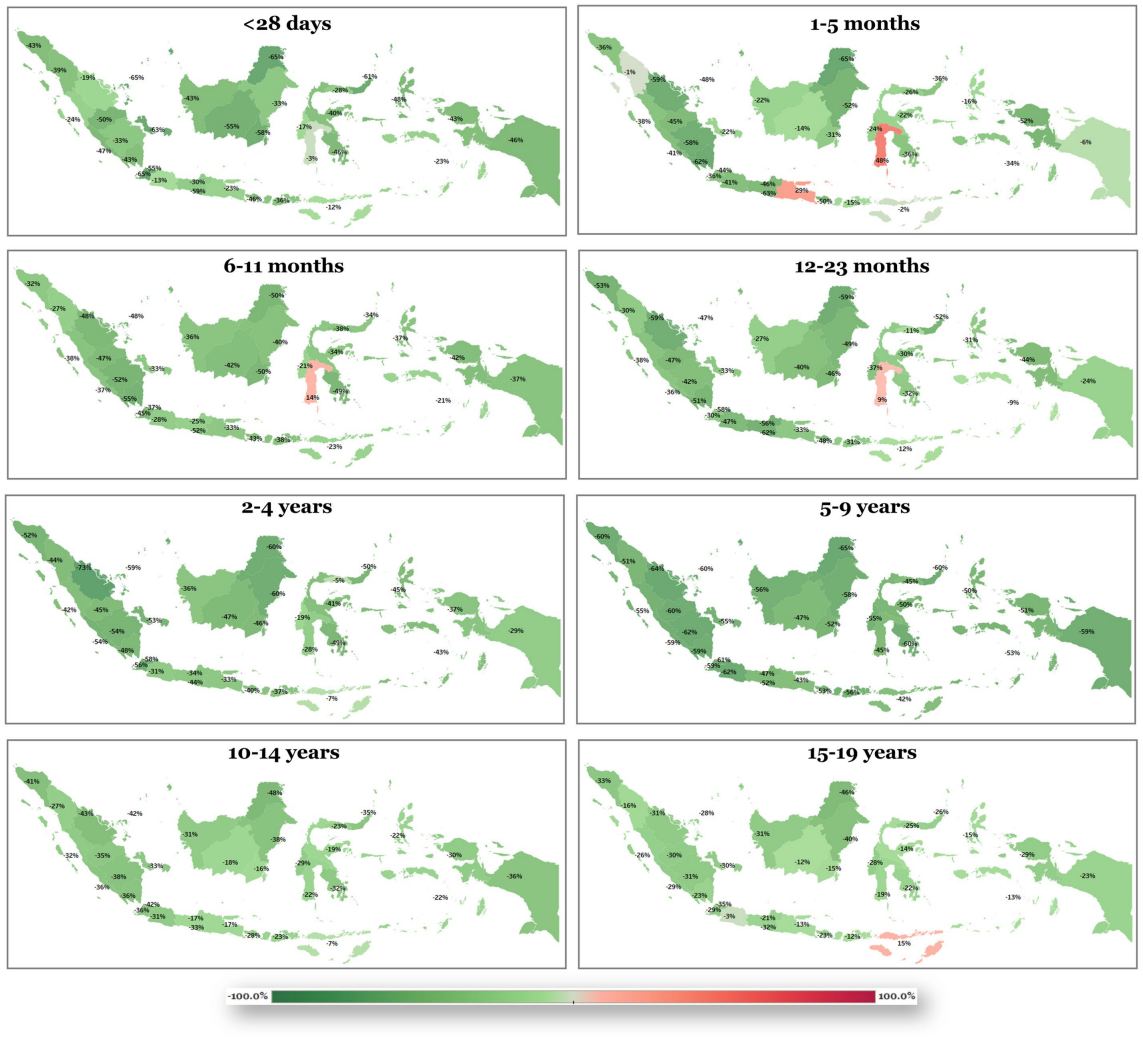
Shift in disability-adjusted life-years (DALYs) for non-congenital cardiovascular diseases (non-congenital CVDs) from 1990 to 2023 across pediatric age groups. Green shading indicates improvement, and red shading indicates worsening.

For the CHA category, prevalence, mortality, and DALY all exhibited increasing trends in the <28 days group (+45.08%, +25.97%, and +91.71%, respectively) (Figure 1). In older age groups, these burdens progressively declined, with the sharpest decreases occurring in the 6–11 months group (−43.79%, −44.38%, and −47.81%, respectively). Among 15–19-year-olds, the reductions were more moderate, with changes of −11.08% in prevalence, −5.95% in mortality, and −11.15% in DALY. On the other hand, among non-congenital CVD category, prevalence showed an overall increasing trend, with the sharpest rise observed in the <28 days age group (+16.34% from 1990 to 2023) (Figure 2). In contrast, mortality declined across all age groups, with the greatest reduction noted in the 6–11 months group (−58.89%). DALY rates increased substantially in the <28 days group (+71.30%), although both mortality and DALY began to decrease from the 1–5 months age group onward. By adolescence (15–19 years), the decline in DALY reached −29.86%, while mortality decreased more prominently (−35.66%).

### 3.4 Shifting burdens based on non-congenital CVD subtypes

An increase in prevalence across all age groups was observed for cardiomyopathy and myocarditis as well as pulmonary heart disease. In contrast, a decline in prevalence from 1990 to 2023 was noted for endocarditis and stroke, although endocarditis showed a localized increase of +10% among the 10–14-year group. RHD demonstrated relatively stable prevalence across all ages, except for a slight decrease of −2% in the 15–19-year group. Meanwhile, IHD and non-rheumatic VHD increased by 33 and 18%, respectively, in 2023 compared to 1990. HHD showed no measurable change (0%) during the same period.

In terms of mortality, nearly all CVD subtypes demonstrated declining trends between 1990 and 2023. Endocarditis and PAH showed reductions of approximately −50% across the 0–9-year age range. The decline was even more pronounced for RHD, with mortality decreasing by roughly −80% within the same age groups. Stroke mortality declined by about −30% among 0–4-year-olds, reaching its greatest reduction (−54%) in the 5–9-year group. However, the decline was more modest in older children and adolescents, with decreases of only −6% in 10–14 years and −14% in 15–19 years. In contrast, mortality due to aortic aneurysm increased substantially by +63%, particularly among females (+89%). IHD and non-rheumatic VHD also showed notable increases in mortality, by +33% and +65%, respectively.

Similar to mortality trends, most CVD subtypes showed negative shifts in DALYs between 1990 and 2023 across all age groups, except for HHD (+21%), IHD (+33%), aortic aneurysm (+63%), and non-rheumatic VHD (+64%). DALYs from cardiomyopathy and myocarditis increased by +26% in the 10–14-year group and +10% in the 15–19-year group, while younger age groups showed mostly negative shifts. Endocarditis, PAH, and stroke demonstrated marked declines, with the largest reductions in the 5–9-year group (−64%, −62%, and −41%, respectively). The greatest DALY reduction was observed in RHD among those aged 12–23 months (−81%), and this decline remained above −50% in older age groups.

### 3.5 Temporal trends of DALY rates

**Figure 3:**
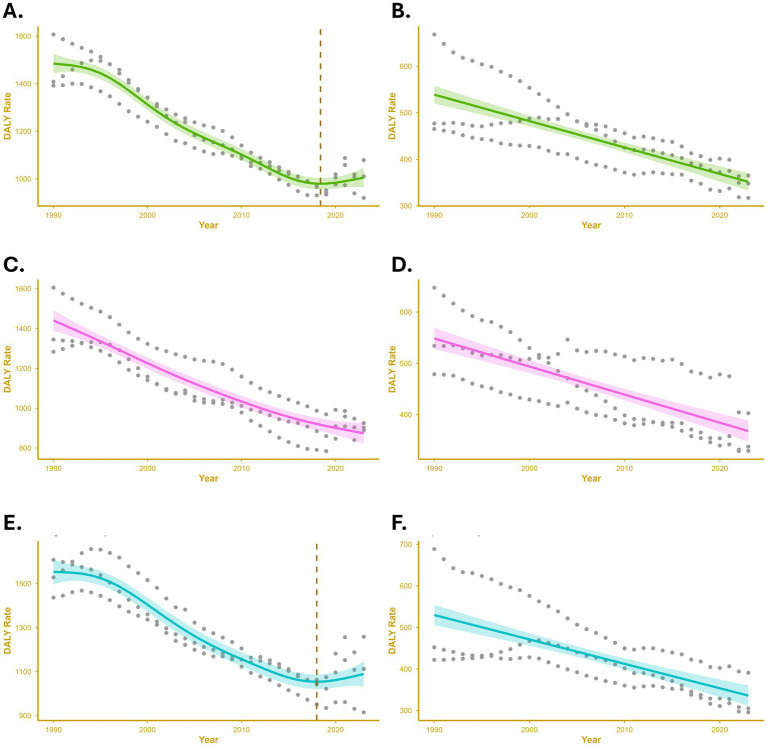
Temporal trends in disability-adjusted life-year (DALY) rates for congenital heart anomalies (CHAs) and non-congenital cardiovascular diseases (non-congenital CVDs) among individuals <20 years of age, modeled using generalized additive models (GAMs). Panels show smoothed temporal trends with shaded 95% confidence intervals: **(A)** CHA, both sexes; **(B)** non-congenital CVDs, both sexes; **(C)** CHA, females; **(D)** non-congenital CVDs, females; **(E)** CHA, males; **(F)** non-congenital CVDs, males. Dashed vertical lines indicate inflection years identified from the GAM smooth terms.

Temporal trends of DALY rates based on the GAM for individuals under 20 years old are presented in Figure 3. A significant non-linear pattern was observed for CHAs, mainly influenced by male trends, with inflection points around 2018 for both sexes combined and for males. In contrast, non-congenital CVDs showed no clear inflection, suggesting more stable or gradual changes over time.

For CHAs, the most pronounced temporal variation occurred during infancy, particularly in those aged 1–5 months, where the model fit was strongest (adjusted *R*^2^ > 0.9), explaining over 90% of the deviance. Early inflection points were identified in the mid-1990s, indicating a historical shift in CHA burden, while more recent changes appeared among neonates around 2018–2020. Beyond early childhood, temporal patterns became more stable, as reflected by lower *R*^2^ values and reduced deviance explained.

For non-congenital CVDs, significant yet less complex temporal variations were detected across all age groups. The best model fits occurred in children aged 5–9 years, particularly among females, where over 90% of the deviance was explained. Moderate nonlinearity emerged in male adolescents, with a subtle inflection around 2000, while infants and older adolescents showed weaker fits and minimal temporal fluctuation. These results indicate that temporal dynamics of non-congenital CVDs were most evident in middle childhood, whereas CHAs displayed sharper, age-dependent variations with earlier and more recent inflection points. The detailed age-specific GAM outputs are summarized in Supplementary Table S7.

### 3.6 Estimated annual percentage changes

The national EAPCs of pediatric CVDs are summarized in Table 3. Both CHAs and non-congenital CVDs showed significant and steady declines, with EAPCs of −1.50 (95% CI: −1.70 to −1.30) and −0.75 (95% CI: −0.93 to −0.57), respectively. Among non-congenital subtypes, endocarditis, RHD, and PAH exhibited the sharpest decreases, with EAPCs of −1.63 (95% CI: −1.70 to −1.56), −2.28 (95% CI: −2.48 to −2.07), and −2.24 (95% CI: −2.39 to −2.09), respectively. In contrast, consistent with the raw DALY changes, aortic aneurysm (0.57 [95% CI: 0.46 to 0.68]), IHD (1.92 [95% CI: 1.53 to 2.31]), and HHD (0.84 [95% CI: 0.59 to 1.08]) showed significant increasing trends (*p* < 0.001).

**Table 3:** National estimated annual percentage change (EAPC) for pediatric cardiovascular diseases (0–19 years) in Indonesia from 1990 to 2023.

**Cause**	**EAPC (95%CI)**	***p*-value**
Congenital heart anomalies (CHAs)	−1.50 (−1.70 to −1.30)	<0.001
Non-congenital cardiovascular diseases (CVDs)	−0.75 (−0.93 to −0.57)	<0.001
Stroke	−0.68 (−0.89 to −0.47)	<0.001
Hypertensive heart disease (HHD)	0.84 (0.59 to 1.08)	<0.001
Cardiomyopathy and myocarditis	−0.81 (−0.95 to −0.67)	<0.001
Aortic aneurysm	0.57 (0.46 to 0.68)	<0.001
Endocarditis	−1.63 (−1.70 to −1.56)	<0.001
Non-rheumatic valvular heart disease (VHD)	0.77 (0.66 to 0.87)	<0.001
Rheumatic heart disease (RHD)	−2.28 (−2.48 to −2.07)	<0.001
Ischemic heart disease (IHD)	1.92 (1.53 to 2.31)	<0.001
Pulmonary Arterial Hypertension (PAH)	−2.24 (−2.39 to −2.09)	<0.001
Others	0.06 (−0.06 to 0.19)	0.32

**Figure 4:**
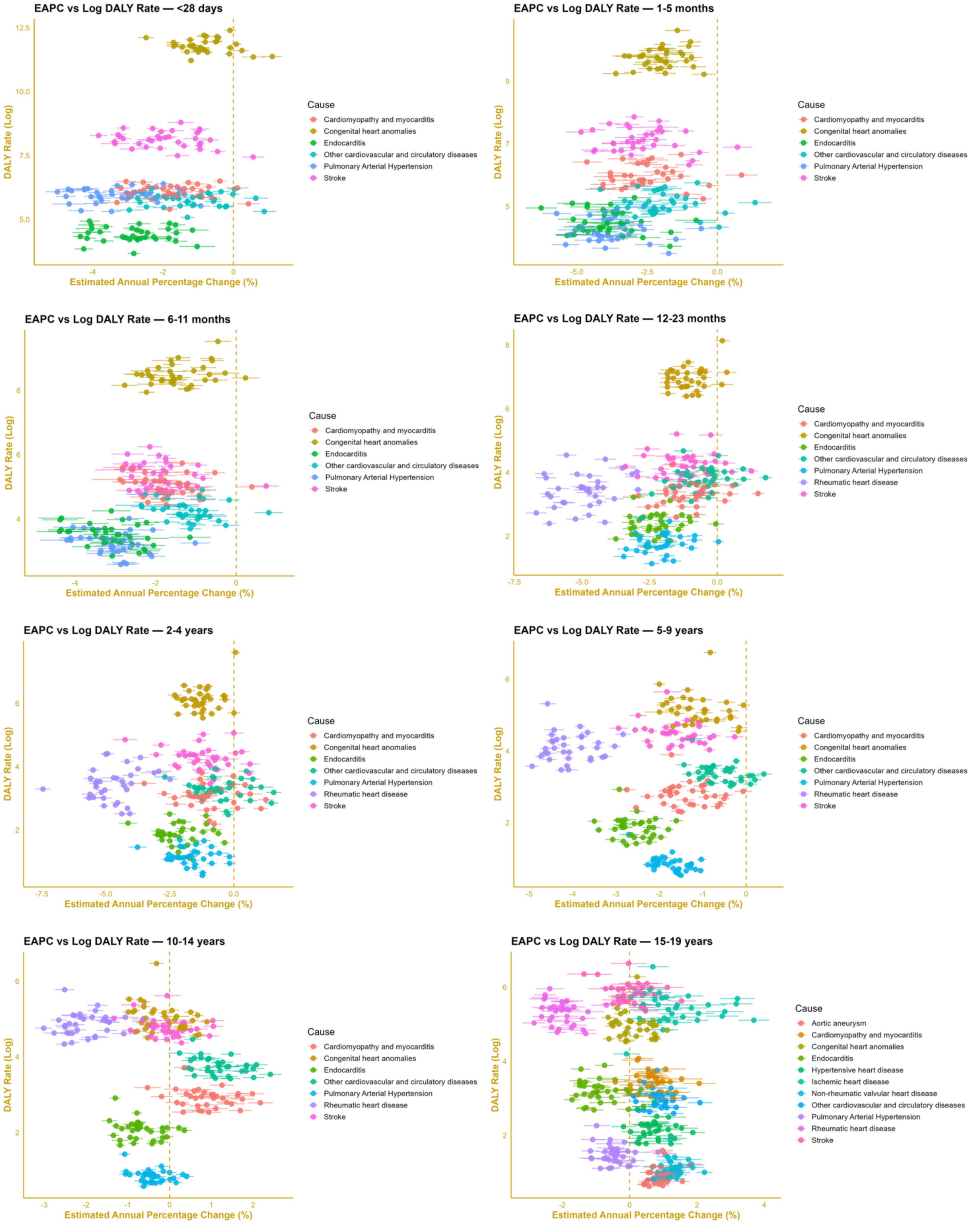
Estimated annual percentage change (EAPC) in disability-adjusted life-years (DALYs) for congenital heart anomalies (CHAs) and non-congenital cardiovascular diseases (non-congenital CVDs) from 1990 to 2021 in pediatric populations across different age groups.

The subnational EAPCs for CHAs and non-congenital CVDs are presented in Figure 4. Among the 0–23-month population, both CHAs and non-congenital CVD subtypes generally showed significant declining trends, except in a few provinces. Significant increases in CHA burden were observed in South Sumatra (*p* = 0.001), South Sulawesi (*p* = 0.017), and North Sumatra (*p* < 0.001). In South Sulawesi, the same age group also demonstrated significant increases in cardiomyopathy and myocarditis, as well as stroke (all *p* < 0.001). East Nusa Tenggara showed positive EAPCs in the 12–23-month group for stroke (*p* < 0.001) and for cardiomyopathy and myocarditis (*p* = 0.003).

More positive EAPCs were observed among the 10–19-year group, where cardiomyopathy and myocarditis tended to increase, while most provinces showed non-significant EAPCs for stroke and CHAs, with no consistent direction of change. In the 10–14-year group, a significant positive change in stroke-related DALY rates was found in Maluku (*p* < 0.001) and North Maluku (*p* = 0.013). Cardiomyopathy and myocarditis in this age group also increased significantly in Maluku (*p* < 0.001), North Maluku (*p* < 0.001), West Papua (*p* < 0.001), and Papua (*p* < 0.005). In contrast, the DALY rate for CHAs declined significantly in West Papua and Maluku among those aged 15–19 years (both *p* < 0.001). In the same age group, most provinces exhibited significantly positive EAPCs for aortic aneurysm, non-rheumatic VHD, IHD, and HHD, which are consistent with the national trend.

### 3.7 Mortality rate trends and regional differences

**Figure 5:**
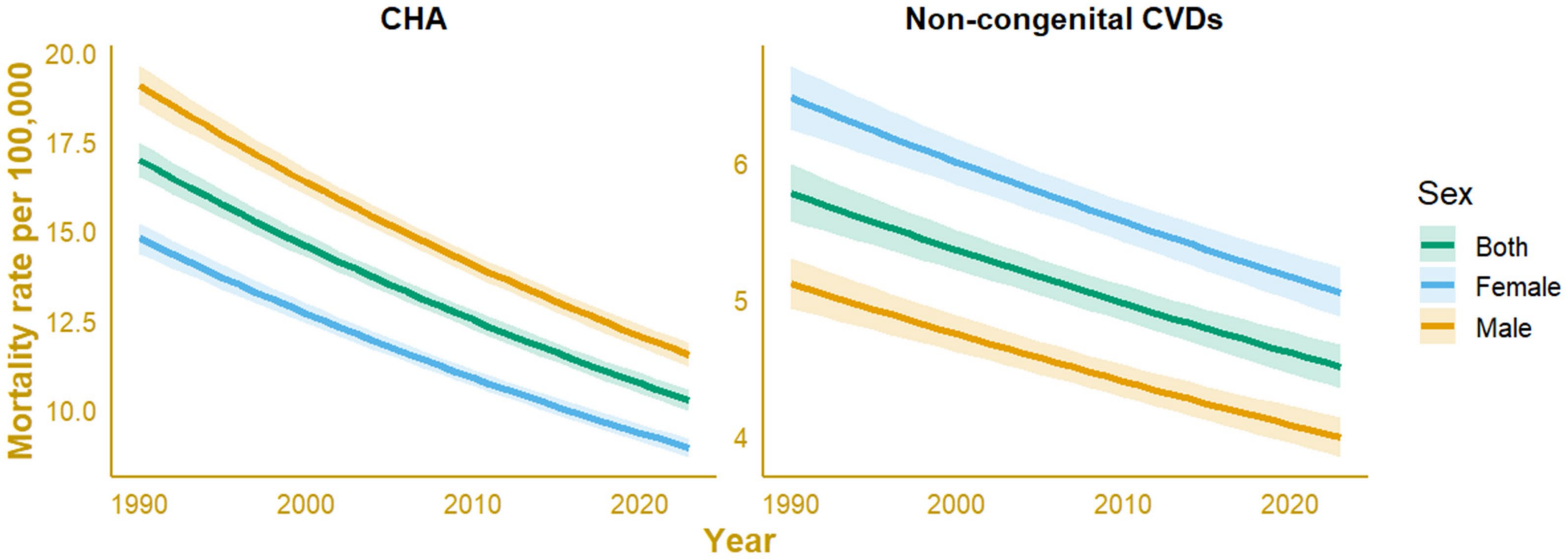
Sex-specific mortality rate trends for congenital heart anomalies (CHAs) and non-congenital cardiovascular diseases (non-congenital CVDs) among the pediatric population (<20 years), modeled using negative binomial regression (NBR).

CHAs and non-congenital CVD-associated mortality rates among pediatric population in Indonesia, between 1990 and 2023, are presented in Figure 5. The trend in mortality show a steady declining number, with overdispersion exceeded 1.5. Negative binomial regression models were applied for both causes due to evidence of overdispersion in the count data. For CHAs, the fitted mortality rate decreased from approximately 16.98 (95% CI: 16.50 to 17.47) deaths per 100,000 in 1990 to 10.26 (95% CI:9.96 to 10.56) deaths per 100,000 in 2023, with male rates consistently higher than those for females. In contrast, non-congenital CVDs showed a more modest decline, from 5.77 (95% CI: 5.57 to 5.98) to 4.51 (95% CI: 4.35 to 4.67) deaths per 100,000 over the same period, where female mortality rates slightly exceeded those of males. Across both causes, the combined-sex category remained intermediate between the sex-specific estimates. The declining trajectories were accompanied by narrow 95% confidence intervals, corresponding to stable model performance and consistent downward trends over time.

**Figure 6:**
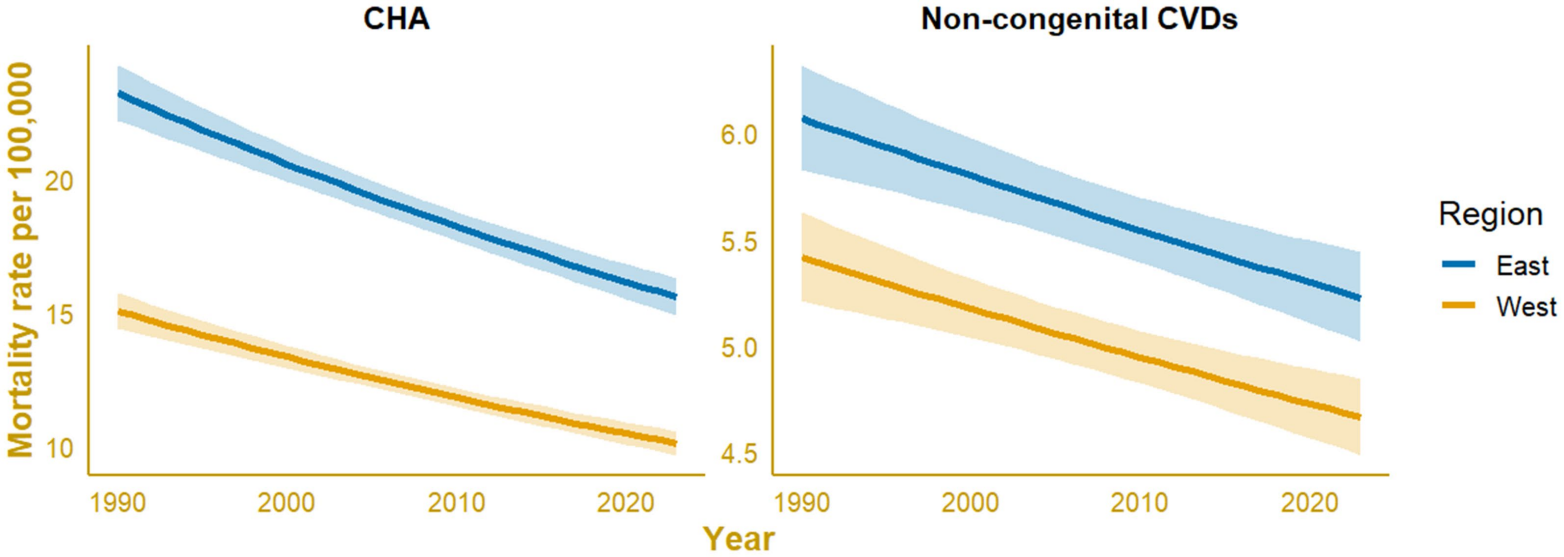
Regional disparities in pediatric mortality trends (<20 years) for congenital heart anomalies (CHAs) and non-cardiovascular diseases (non-CVDs) in Indonesia from 1990 to 2023, modeled using negative binomial regression (NBR).

Regional modeling revealed persistent geographic disparities in pediatric mortality across Indonesia. Negative binomial regression models were applied because the overdispersion for both causes exceeded 1.5. Both CHAs and non-congenital CVDs showed steady declines in mortality rates from 1990 to 2023 in both the western and eastern regions (Figure 6). The eastern region consistently exhibited higher mortality rates throughout the period, with slower decline. For CHAs, mortality decreased from 23.20 (95% CI: 22.18 to 24.26) to 15.58 (95% CI: 14.90 to 16.30) deaths per 100,000 in the East and from 15.06 (95% CI: 14.41 to 15.74) to 10.12 (95% CI: 9.68 to 10.58) per 100,000 in the West. Similarly, non-congenital CVD mortality declined from 6.07 (95% CI: 5.83 to 6.32) to 5.23 (95% CI: 5.02 to 5.44) per 100,000 in the East and from 5.41(95% CI: 5.21 to 5.63) to 4.66 (95% CI: 4.48 to 4.85) per 100,000 in the West.

### 3.8 Model consistency

**Figure 7:**
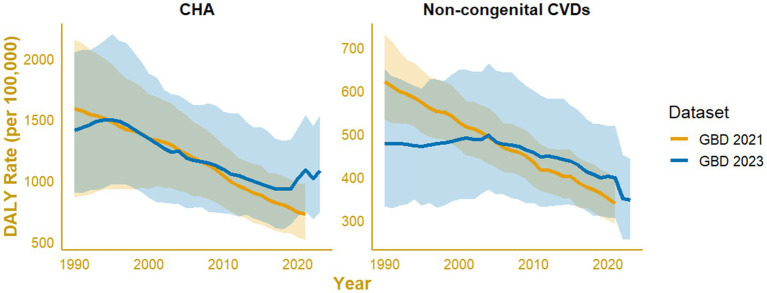
Comparison of generalized additive model (GAM)–fitted disability-adjusted life-year (DALY) rate trends between Global Burden of Disease (GBD) 2021 and GBD 2023 datasets for individuals under 20 years of age in Indonesia. Panels show fitted smooth curves with shaded 95% confidence intervals for non-cardiovascular diseases (non-CVD; left) and congenital heart anomalies (CHAs; right).

The sensitivity analysis demonstrated high consistency in temporal patterns and model performance between the GBD 2021 and GBD 2023 datasets (Figure 7). For the GBD 2021 data, the models showed near-perfect fits, with adjusted R^2^ values of 0.998 for non-congenital CVDs and 0.999 for CHAs, explaining over 99% of the deviance. Similar results were obtained for GBD 2023, with adjusted R^2^ values of 0.972 and 0.990, and deviance explained of 97.8 and 99.3% for non-congenital CVDs and CHAs, respectively. The effective degrees of freedom (edf = 6.2–7.9) indicated mild nonlinearity in both datasets, while the overall trend structures remained consistent. The robustness of the observed trends is shown in comparratively fitted smooth curves, where both GBD releases produced highly concordant temporal trajectories (Figure 7).

## 4 Discussion

In the present study, CHAs were identified as the leading contributor to the burden of overall pediatric CVDs in Indonesia. The highest prevalence and mortality rates for CHAs were observed among neonates under 28 days old, at 1511.53 per 100,000 and 1341.68 per 100,000, respectively, with the highest DALY rate reaching 120,809.85 per 100,000. Nationally, declining trends in disease burden were observed, although the trend appeared stagnant among adolescents. Declining trends in the burden of CHAs among neonates likely reflects a combination of factors, including the increasing number of pediatric cardiologists, obstetricians, and cardiac surgeons in Indonesia (10); greater community awareness and health literacy supported by online information sources (20); and national health initiatives—such as the Ministry of Health’s distribution of pulse oximetry and portable echocardiography devices for early detection of CHAs and RHD in remote areas (20, 21). The observed decline in prevalence and mortality in younger groups likely reflects the cumulative impact of national health strategies such as expanded immunization, improved antenatal care, and strengthened neonatal and maternal services (10, 22). However, the persistence of CHA burden, particularly in older children, may reflect gaps in long-term management, limited access to pediatric cardiac surgery, and barriers to timely diagnosis and follow-up care. Improved detection at the primary care level is essential, with general practitioners playing a pivotal role in recognizing murmurs and other signs of CHAs during routine examinations.

Screening efforts, such as those led by cardiologists across Indonesian provinces, have improved documentation and detection, but regional disparities remain, especially in Eastern Indonesia (23–25). Notably, the EAPC analysis in the present study revealed positive trends for CHAs in specific provinces, such as South Sumatra, South Sulawesi, and North Sumatra. Our analysis also showed that western provinces experienced more improvement in disease burden than eastern ones. In western provinces people have less access to skilled health personnel, adequately equipped health centers, and a lower likelihood of utilizing insurance coverage (26). In a cross-sectional study, 46.6% of the total respondents from Papua, one of Indonesia’s western provinces, reported travel times of more than 1 h to reach hospitals, the highest across all regions surveyed (26). A study reported that women residing in eastern provinces, including Papua and West Papua, have markedly lower odds of attending the recommended four or more antenatal care visits compared to those in more developed regions like Java and Bali (27). In another study, factors such as limited healthcare infrastructure, shortages of skilled health personnel, and challenging geographical terrains are attributed to lower odds of utilizing antenatal care and maternal services in Papua compared to women in Java and Bali (28).

In this study, both congenital and non-congenital CVDs exhibited distinct but interconnected transitions across childhood and adolescence. For CHAs, positive EAPC values among neonates were found mainly in Sumatra provinces in the west and South Sulawesi in the east. As survival improved with age, the CHA burden shifted toward older pediatric populations, with positive EAPC values also appearing in wealthier regions such as Jakarta, Yogyakarta, and Bali, where advanced facilities enable early diagnosis and longer survival, thereby increasing years lived with disability. However, increasing burdens of adolescents in eastern may be attributable to mortality, as shown in our present study. Similar transitions were observed among non-congenital CVDs, with the rising trend in South Sulawesi and East Nusa Tenggara and persistent stroke impact among adolescents. Limited access to timely diagnosis, referral facilities, and interventional cardiologists may explain the slower improvement observed in eastern provinces. Nonetheless, the growing exposure to behavioral and metabolic risks (such as unhealthy diet, physical inactivity, and tobacco use) appears to contribute across regions, regardless of geospatial differences (29–31). Collectively, these findings depict a shifting epidemiology where congenital and non-congenital conditions coexist and interact across developmental stages. This underscores the increasing need for integrated, age-specific cardiovascular services that connect pediatric and adult care (32). Although Indonesia has strengthened its cardiac care capacity through expanded training, improved technology, and minimally invasive interventions such as zero-fluoroscopy defect closure and CHA stenting (33), access remains inequitable. Socioeconomic factors, including poverty, parental education, and health literacy, continue to widen the gap between survival-rich and mortality-dominated regions, as previously reported (34, 35).

The rising cardiomyopathy and myocarditis burden among adolescents, along with the stagnating decline in RHD, further underscores the unmet needs in continuity of care (10, 36, 37). In addition, IHD, HHD, and aortic aneurysm showed positive EAPC values, indicating increasing burdens that signal the early emergence of adult-type cardiovascular conditions within younger age groups. These trends suggest that the adolescent population is entering a transition phase where congenital, acquired, and degenerative cardiovascular pathologies overlap. Yet, this group remains largely underrepresented in national surveillance, leading to underestimation of their true burden. Strengthening longitudinal monitoring systems and developing adolescent cardiac registries are therefore crucial to uncovering these hidden patterns and guiding more targeted prevention strategies. Currently, the largest registry for pediatric CVD has only been established in a single center in Yogyakarta (38).

The present study offers a unique contribution by providing the most up-to-date, multi-decade analysis of pediatric cardiovascular burdens in Indonesia using GBD 2023 data, integrating national, provincial, and age-specific modeling. Furthermore, it provides valuable insights into persistent east–west disparities, revealing slower progress in eastern provinces. By applying GAM and EAPC analyses, this work captures both linear and nonlinear temporal shifts that conventional methods might overlook, offering a more nuanced understanding of age-transition dynamics. These methodological strengths provide a foundation for policy translation, particularly for monitoring emerging risks among adolescents, a population often masked within broader child or adult datasets. Strengthening adolescent cardiac surveillance, expanding registry coverage beyond Yogyakarta, and embedding cardiovascular screening into school health programs should be prioritized.

This study has several strengths. First, it leverages three decades of nationwide data, providing a long-term view of pediatric cardiovascular disease patterns across Indonesia. Second, the use of province-level granularity allows for detailed assessment of geographical inequalities, offering insights that would not be visible in national-level summaries alone. Third, the application of standardized GBD case definitions, DALYs, and EAPC enables consistent comparisons across time, regions, and disease categories.

Nonetheless, several limitations should be considered when interpreting the findings. All estimates derive from the GBD study, which synthesizes heterogeneous data sources and may introduce bias due to variability in the completeness and quality of provincial health records, underreporting, and inconsistencies in diagnostic or coding practices. As a result, the findings are subject to the constraints of the underlying data, including provincial differences in data quality and the absence of individual-level verification. Provinces with limited health information systems may be particularly affected by underreporting, potentially leading to underestimated disease burdens. The absence of uncertainty intervals for provincial estimates limits assessment of precision, and changes in GBD estimation methods across cycles may introduce artefacts affecting temporal trends. The ecological study design also precludes causal inference and restricts examination of individual-level determinants, such as parental health history, socioeconomic conditions, or environmental exposures. Moreover, as GBD estimates are model-based, they may not fully capture the impact of recent local interventions or improvements in pediatric cardiac care implemented after the last available input data. Additionally, the GBD database is designed to provide population-level modeled estimates rather than individual-level data, which limits the capacity to explore clinical or household determinants, such as parental health history, socioeconomic factors, or environmental exposures. Future studies incorporating primary data collection or linkage with individual-level records would be necessary to validate and expand upon the patterns reported here.

## 5 Conclusion

Despite meaningful national declines in pediatric CVD burden over the past three decades, persistent provincial disparities threaten equitable cardiovascular outcomes for Indonesian children. These findings highlight that progress has been uneven, with eastern provinces continuing to experience higher mortality and more limited access to pediatric cardiac services. The substantial burden of CHAs among neonates and the rising adolescent trends for adult-type cardiovascular conditions indicate gaps in long-term management and transition-of-care pathways. Ensuring more equitable distribution of diagnostic capacity, specialist services, and referral networks is essential to close these gaps. Strengthening adolescent cardiovascular surveillance, expanding provincial registry systems, and integrating cardiovascular risk prevention into school and primary care programs represent key steps to sustain early survival gains and support healthier trajectories into adulthood.

## Data Availability

Publicly available datasets were analyzed in this study. The data used in this study are publicly available from the Global Burden of Disease (GBD) portal and can be accessed at http://ghdx.healthdata.org/gbd-results-tool.

## Footnotes

1
http://ghdx.healthdata.org/gbd-results-tool
