# Supplementary material for: Geographical inequalities and temporal trends in pediatric cardiovascular diseases in Indonesia: a 34-year global burden of disease analysis
Source: Front Public Health. 2025 Dec 16;13:1688700. doi: 10.3389/fpubh.2025.1688700 (PMC12748149; doi:10.3389/fpubh.2025.1688700)
Supplement: Supplementary file 1 [file Supplementary_file_1.docx]

Table S1. Prevalence and its shift from 1990 to 2023 of pediatric cardiovascular diseases among populations aged <28 days to 23 months

| Disease sub-category | < 28 days | | 1 – 5 months | | 6 – 11 months | | 12 – 23 months | |
| --- | --- | --- | --- | --- | --- | --- | --- | --- |
|  | Preval (95% UI) | Δ (%) | Preval (95% UI) | Δ (%) | Preval (95% UI) | Δ (%) | Preval (95% UI) | Δ (%) |
| Cardiovascular diseases |  |  |  |  |  |  |  |  |
| Both | 6.21 (8.8-4.62) | 10 | 33.28 (43.89-25.96) | 9 | 100.35 (129.97-78.19) | 7 | 121.34 (148.38-100.71) | 11 |
| Female | 6.79 (9.86-4.99) | 16 | 34.58 (45.02-26.79) | 15 | 102.43 (132.28-78.69) | 14 | 113.76 (137.77-94.67) | 12 |
| Male | 5.66 (7.81-4.28) | 4 | 32.04 (41.66-24.89) | 3 | 98.39 (131.4-75.96) | 1 | 128.53 (158.97-104.53) | 10 |
| Congenital heart anomalies |  |  |  |  |  |  |  |  |
| Both | 1511.53 (1806.59-1247.34) | -5 | 1011.83 (1190.15-844.54) | -4 | 740.05 (877.99-618.22) | -4 | 617.44 (725.39-519.26) | -3 |
| Female | 1453.99 (1734.94-1204.54) | -5 | 985.91 (1164.63-821.19) | -5 | 731.08 (872.31-609.46) | -4 | 608.92 (728.59-507.58) | -4 |
| Male | 1565.95 (1868.41-1284.02) | -5 | 1036.37 (1220.2-870.33) | -4 | 748.54 (889.59-622.67) | -4 | 625.53 (729.71-526.92) | -3 |
| Cardiomyopathy and myocarditis |  |  |  |  |  |  |  |  |
| Both | 0.99 (1.45-0.68) | 14 | 6.05 (8.71-4.09) | 22 | 12.94 (18.17-9.24) | 26 | 19.18 (26.16-13.55) | 31 |
| Female | 1.02 (1.5-0.68) | 8 | 6.34 (9.24-4.32) | 10 | 13.64 (19.09-9.92) | 16 | 18.97 (26.63-13.22) | 22 |
| Male | 0.96 (1.41-0.66) | 21 | 5.78 (8.38-4) | 37 | 12.28 (17.44-8.85) | 39 | 19.38 (26.52-13.82) | 42 |
| Endocarditis |  |  |  |  |  |  |  |  |
| Both | 0.13 (0.18-0.1) | -25 | 0.49 (0.6-0.39) | -33 | 1.08 (1.34-0.87) | -37 | 1.6 (2-1.27) | -21 |
| Female | 0.13 (0.17-0.09) | -18 | 0.5 (0.62-0.41) | -17 | 1.32 (1.66-1.05) | -19 | 1.82 (2.3-1.42) | 20 |
| Male | 0.14 (0.19-0.1) | -30 | 0.47 (0.58-0.38) | -44 | 0.86 (1.04-0.69) | -52 | 1.39 (1.72-1.1) | -45 |
| Pulmonary arterial hypertension |  |  |  |  |  |  |  |  |
| Both | 0.01 (0.01-0) | 15 | 0.05 (0.07-0.03) | 14 | 0.11 (0.15-0.07) | 13 | 0.17 (0.24-0.12) | 12 |
| Female | 0.01 (0.01-0) | 8 | 0.05 (0.07-0.03) | 8 | 0.11 (0.16-0.08) | 7 | 0.18 (0.26-0.13) | 5 |
| Male | 0.01 (0.01-0) | 22 | 0.04 (0.06-0.03) | 22 | 0.1 (0.14-0.07) | 21 | 0.16 (0.23-0.11) | 21 |
| Rheumatic heart disease |  |  |  |  |  |  |  |  |
| Both | ND | ND | ND | ND | ND | ND | 4.94 (7.16-3.12) | 0 |
| Female | ND | ND | ND | ND | ND | ND | 4.78 (6.94-3.05) | 1 |
| Male | ND | ND | ND | ND | ND | ND | 5.08 (7.42-3.18) | 0 |
| Stroke |  |  |  |  |  |  |  |  |
| Both | 1.75 (2.04-1.52) | -12 | 9.15 (10.53-7.92) | -11 | 20.76 (23.68-18.01) | -11 | 38.29 (43.54-33.05) | -11 |
| Female | 1.9 (2.19-1.67) | -9 | 9.93 (11.48-8.62) | -8 | 22.53 (25.79-19.52) | -8 | 41.5 (47.06-35.97) | -8 |
| Male | 1.61 (1.9-1.37) | -15 | 8.41 (9.81-7.25) | -14 | 19.08 (22.07-16.37) | -13 | 35.24 (40.28-30.16) | -14 |
| Others |  |  |  |  |  |  |  |  |
| Both | 3.33 (5.72-1.78) | 28 | 17.55 (27.36-11.36) | 21 | 65.5 (96.38-44.03) | 12 | 57.21 (83.07-38.02) | 28 |
| Female | 3.73 (6.73-1.94) | 42 | 17.77 (28.34-11.18) | 38 | 64.86 (97.99-42.57) | 25 | 46.55 (70.76-29.81) | 36 |
| Male | 2.94 (4.97-1.59) | 14 | 17.34 (27.23-10.97) | 7 | 66.09 (98.43-42.97) | 3 | 67.32 (99.16-46.19) | 24 |

ND, no data; (Δ), prevalence shift from 1990 to 2021.

Table S2. Prevalence and its shift from 1990 to 2021 of pediatric cardiovascular diseases among populations aged 2 to 19 years

| Disease sub-category | 2-4 years | | 5-9 years | | 10-14 years | | 15-19 years | |
| --- | --- | --- | --- | --- | --- | --- | --- | --- |
|  | Preval (95% UI) | Δ (%) | Preval (95% UI) | Δ (%) | Preval (95% UI) | Δ (%) | Preval (95% UI) | Δ (%) |
| Cardiovascular diseases |  |  |  |  |  |  |  |  |
| Both | 21.41 (29.34-14.94) | 10 | 400.16 (476.77-329.15) | 8 | 619.7 (745.48-501.99) | 9 | 831.49 (966.86-716.33) | 0 |
| Female | 20.81 (28.8-14.47) | 12 | 404.85 (485.05-334.77) | 10 | 601.02 (725.38-495.66) | 9 | 839.14 (977.99-728.46) | -1 |
| Male | 21.98 (29.93-15.38) | 7 | 395.66 (469.74-319.11) | 6 | 637.37 (788.31-506.09) | 8 | 824.33 (965.79-705.21) | 0 |
| Congenital heart anomalies |  |  |  |  |  |  |  |  |
| Both | 417.31 (478.6-356.31) | -1 | 218.19 (251.6-190.67) | 0 | 164.65 (191.21-141.1) | 0 | 151.42 (174.24-130.67) | 1 |
| Female | 400.72 (465.99-344.19) | -2 | 204.55 (232.8-180.21) | -1 | 162 (185.84-140.39) | 1 | 152.45 (173.62-131.88) | 2 |
| Male | 433.11 (494.58-370.03) | 1 | 231.24 (271.43-199.98) | 2 | 167.16 (195.91-142.18) | 0 | 150.45 (174.81-128.02) | 0 |
| Cardiomyopathy and myocarditis |  |  |  |  |  |  |  |  |
| Both | 21.41 (29.34-14.94) | 53 | 18.04 (28.05-11.3) | 42 | 17.82 (26.86-11.86) | 48 | 22.97 (34.2-14.23) | 53 |
| Female | 20.81 (28.8-14.47) | 41 | 15.76 (24.24-10) | 38 | 15.11 (22.85-10.06) | 50 | 18.87 (28.94-11.44) | 63 |
| Male | 21.98 (29.93-15.38) | 67 | 20.22 (31.74-12.45) | 46 | 20.4 (30.69-13.3) | 47 | 26.81 (38.96-16.84) | 45 |
| Endocarditis |  |  |  |  |  |  |  |  |
| Both | 2.19 (2.81-1.69) | -12 | 1.75 (2.4-1.19) | -8 | 1.11 (1.52-0.78) | 10 | 2.25 (3.41-1.43) | 5 |
| Female | 2.43 (3.13-1.86) | 21 | 1.6 (2.22-1.1) | 32 | 1.04 (1.41-0.74) | 39 | 2.29 (3.47-1.47) | 32 |
| Male | 1.97 (2.48-1.52) | -34 | 1.9 (2.61-1.29) | -26 | 1.18 (1.63-0.82) | -6 | 2.21 (3.31-1.41) | -13 |
| Hypertensive heart disease |  |  |  |  |  |  |  |  |
| Both | ND | ND | ND | ND | ND | ND | 2.54 (4.25-1.48) | 0 |
| Female | ND | ND | ND | ND | ND | ND | 2.6 (4.33-1.47) | 1 |
| Male | ND | ND | ND | ND | ND | ND | 2.47 (4.13-1.46) | -1 |
| Ischemic heart disease |  |  |  |  |  |  |  |  |
| Both | ND | ND | ND | ND | ND | ND | 96.33 (136.07-63.58) | 33 |
| Female | ND | ND | ND | ND | ND | ND | 102.35 (143.94-68.58) | 20 |
| Male | ND | ND | ND | ND | ND | ND | 90.68 (129.04-57.66) | 52 |
| Non-rheumatic valvular heart disease |  |  |  |  |  |  |  |  |
| Both | ND | ND | ND | ND | ND | ND | 0.1 (0.13-0.07) | 18 |
| Female | ND | ND | ND | ND | ND | ND | 0.1 (0.13-0.07) | 20 |
| Male | ND | ND | ND | ND | ND | ND | 0.1 (0.14-0.07) | 17 |
| Pulmonary arterial hypertension |  |  |  |  |  |  |  |  |
| Both | 0.27 (0.36-0.19) | 12 | 0.37 (0.51-0.27) | 13 | 0.51 (0.76-0.33) | 15 | 0.73 (1.17-0.41) | 17 |
| Female | 0.29 (0.4-0.21) | 4 | 0.41 (0.55-0.29) | 4 | 0.55 (0.8-0.35) | 4 | 0.78 (1.23-0.44) | 5 |
| Male | 0.24 (0.33-0.17) | 22 | 0.34 (0.46-0.24) | 26 | 0.48 (0.72-0.3) | 30 | 0.69 (1.11-0.38) | 33 |
| Rheumatic heart disease |  |  |  |  |  |  |  |  |
| Both | 24.05 (35.37-15.29) | 0 | 81.81 (118.02-52.91) | 0 | 162.08 (252.29-96.22) | 0 | 310.9 (464.63-209.01) | -2 |
| Female | 23.33 (34.61-14.96) | 1 | 78.66 (112.76-50.39) | 1 | 154.56 (242.16-93.19) | 1 | 310.73 (464.61-211.15) | -1 |
| Male | 24.74 (36.15-15.46) | 0 | 84.83 (123.54-55.29) | 0 | 169.2 (263.82-99.95) | -2 | 311.07 (467.52-209.94) | -3 |
| Stroke |  |  |  |  |  |  |  |  |
| Both | 80.01 (91.24-70.12) | -12 | 148.29 (173.31-126.18) | -14 | 226.22 (261.52-191.93) | -15 | 316.07 (365.36-269.71) | -14 |
| Female | 86.57 (98.28-75.91) | -10 | 159.54 (187.12-136.12) | -13 | 241.95 (281-206.87) | -15 | 335.05 (385.38-289.19) | -15 |
| Male | 73.78 (84.68-64.33) | -14 | 137.53 (159.57-115.36) | -16 | 211.34 (247.95-179.73) | -15 | 298.28 (344.35-252.14) | -13 |
| Others |  |  |  |  |  |  |  |  |
| Both | 64.44 (91.57-42.91) | 47 | 150.43 (225.15-93.11) | 49 | 213.27 (313.33-134.31) | 65 | 82.01 (143.37-47.19) | 39 |
| Female | 60.79 (86.77-41.17) | 65 | 149.44 (221.85-90.99) | 62 | 189.04 (277.94-122.13) | 85 | 68.79 (113.86-37.49) | 76 |
| Male | 67.9 (97.48-44.35) | 35 | 151.38 (224.17-93.9) | 39 | 236.18 (361.47-145.91) | 52 | 94.41 (162.92-54.14) | 19 |

ND, no data; (Δ), prevalence shift from 1990 to 2021

Table S3. Mortality rate and its shift from 1990 to 2023 of pediatric cardiovascular diseases among populations aged <28 days to 23 months

| Disease sub-category | < 28 days | | 1 – 5 months | | 6 – 11 months | | 12 – 23 months | |
| --- | --- | --- | --- | --- | --- | --- | --- | --- |
|  | Death (95% UI) | Δ (%) | Death (95% UI) | Δ (%) | Death (95% UI) | Δ (%) | Death (95% UI) | Δ (%) |
| Cardiovascular diseases |  |  |  |  |  |  |  |  |
| Both | 39.64 (57.47-25.71) | -34 | 18.35 (28.13-10.84) | -31 | 4.58 (7.16-2.57) | -36 | 1.62 (2.4-0.96) | -43 |
| Female | 43.13 (69.39-25.13) | -36 | 19.53 (33.08-10.12) | -30 | 5.24 (9.82-2.35) | -35 | 1.77 (3.09-0.89) | -54 |
| Male | 36.33 (57.99-17.89) | -32 | 17.23 (30.35-8.21) | -32 | 3.96 (6.8-1.75) | -36 | 1.48 (2.44-0.73) | -22 |
| Congenital heart anomalies |  |  |  |  |  |  |  |  |
| Both | 1341.68 (1989-844.75) | -13 | 174.08 (249.08-112.76) | -23 | 48.93 (74.23-30.46) | -21 | 10.38 (15.55-6.17) | -22 |
| Female | 1051.57 (1743.09-495.75) | -23 | 146.65 (247.96-68.96) | -24 | 43.84 (74.04-20.9) | -31 | 9.02 (15.12-4.19) | -41 |
| Male | 1616.05 (2750.68-799.02) | -6 | 200.06 (330.63-107.22) | -22 | 53.76 (99.41-28.94) | -11 | 11.66 (20.08-6.07) | 3 |
| Cardiomyopathy and myocarditis |  |  |  |  |  |  |  |  |
| Both | 4.43 (9.7-1.51) | -24 | 4.1 (7.89-1.58) | -23 | 1.55 (2.81-0.66) | -30 | 0.28 (0.54-0.11) | -23 |
| Female | 4.47 (9.95-1.21) | -16 | 3.96 (9.33-0.97) | -15 | 1.69 (3.91-0.46) | -25 | 0.32 (0.77-0.09) | -33 |
| Male | 4.4 (14.45-1.08) | -30 | 4.23 (10.24-1.22) | -29 | 1.42 (2.85-0.5) | -34 | 0.24 (0.58-0.08) | -5 |
| Endocarditis |  |  |  |  |  |  |  |  |
| Both | 0.75 (1.76-0.3) | -50 | 0.81 (1.64-0.31) | -54 | 0.28 (0.54-0.11) | -58 | 0.09 (0.2-0.04) | -52 |
| Female | 0.59 (1.34-0.19) | -36 | 0.72 (1.66-0.18) | -43 | 0.33 (0.82-0.08) | -53 | 0.1 (0.24-0.03) | -55 |
| Male | 0.91 (2.35-0.27) | -56 | 0.9 (2.45-0.24) | -60 | 0.23 (0.54-0.07) | -64 | 0.08 (0.2-0.02) | -47 |
| Pulmonary arterial hypertension |  |  |  |  |  |  |  |  |
| Both | 2.91 (8.47-0.49) | -53 | 0.6 (1.7-0.13) | -48 | 0.21 (0.67-0.05) | -54 | 0.04 (0.13-0.01) | -50 |
| Female | 3.51 (10.88-0.53) | -51 | 0.65 (2.11-0.09) | -44 | 0.23 (0.84-0.03) | -53 | 0.05 (0.18-0.01) | -57 |
| Male | 2.33 (9.09-0.17) | -55 | 0.56 (1.98-0.07) | -52 | 0.19 (0.72-0.03) | -56 | 0.04 (0.14-0.01) | -37 |
| Rheumatic heart disease |  |  |  |  |  |  |  |  |
| Both | ND | ND | ND | ND | ND | ND | 0.16 (0.31-0.07) | -81 |
| Female | ND | ND | ND | ND | ND | ND | 0.18 (0.43-0.05) | -85 |
| Male | ND | ND | ND | ND | ND | ND | 0.14 (0.29-0.04) | -72 |
| Stroke |  |  |  |  |  |  |  |  |
| Both | 28.11 (44.32-17.84) | -34 | 10.96 (18.35-6.07) | -33 | 1.81 (3.37-0.94) | -38 | 0.56 (0.94-0.31) | -34 |
| Female | 31.61 (52.21-15.96) | -38 | 12.43 (23.7-5.83) | -35 | 2.19 (4.57-0.82) | -42 | 0.64 (1.15-0.28) | -48 |
| Male | 24.81 (43.73-12.22) | -29 | 9.58 (18.6-4.45) | -30 | 1.44 (2.87-0.59) | -32 | 0.5 (0.97-0.22) | -5 |
| Others |  |  |  |  |  |  |  |  |
| Both | 3.43 (6.63-1.51) | -9 | 1.87 (3.53-0.79) | -6 | 0.73 (1.43-0.32) | -16 | 0.49 (0.82-0.23) | -7 |
| Female | 2.96 (7.9-0.9) | 1 | 1.78 (4.73-0.42) | 0 | 0.8 (1.97-0.21) | -13 | 0.48 (1.09-0.15) | -23 |
| Male | 3.88 (8.37-1.25) | -15 | 1.96 (4.43-0.61) | -11 | 0.67 (1.42-0.21) | -19 | 0.49 (0.93-0.18) | 15 |

ND, no data; (Δ), prevalence shift from 1990 to 2021.

Table S4. Mortality rate and its shift from 1990 to 2021 of pediatric cardiovascular diseases among populations aged 2 to 19 years

| Disease sub-category | 2-4 years | | 5-9 years | | 10-14 years | | 15-19 years | |
| --- | --- | --- | --- | --- | --- | --- | --- | --- |
|  | Death (95% UI) | Δ (%) | Death (95% UI) | Δ (%) | Death (95% UI) | Δ (%) | Death (95% UI) | Δ (%) |
| Cardiovascular diseases |  |  |  |  |  |  |  |  |
| Both | 1.1 (1.64-0.63) | -46 | 1.05 (1.52-0.66) | -63 | 2.29 (3.2-1.53) | -32 | 8.15 (11.08-5.7) | -19 |
| Female | 1.14 (1.92-0.57) | -49 | 1.25 (2.01-0.63) | -66 | 2.53 (3.94-1.43) | -37 | 10.13 (15.64-6.56) | -1 |
| Male | 1.06 (1.83-0.52) | -43 | 0.85 (1.47-0.45) | -59 | 2.07 (3.32-1.18) | -24 | 6.29 (9.88-3.51) | -36 |
| Congenital heart anomalies |  |  |  |  |  |  |  |  |
| Both | 3.9 (5.74-2.44) | -32 | 1.57 (2.33-0.99) | -29 | 1.73 (2.55-1.17) | -3 | 1.73 (2.52-1.16) | 5 |
| Female | 3.1 (5.15-1.52) | -48 | 1.33 (2.09-0.71) | -43 | 1.4 (2.19-0.81) | -19 | 1.54 (2.48-0.83) | -1 |
| Male | 4.66 (8.06-2.57) | -15 | 1.8 (3.13-0.9) | -14 | 2.05 (3.3-1.16) | 11 | 1.91 (3.07-1.08) | 9 |
| Aortic aneurysm |  |  |  |  |  |  |  |  |
| Both | ND | ND | ND | ND | ND | ND | 0.02 (0.04-0.01) | 63 |
| Female | ND | ND | ND | ND | ND | ND | 0.03 (0.05-0.01) | 89 |
| Male | ND | ND | ND | ND | ND | ND | 0.02 (0.05-0.01) | 41 |
| Cardiomyopathy and myocarditis |  |  |  |  |  |  |  |  |
| Both | 0.19 (0.34-0.08) | -19 | 0.12 (0.21-0.05) | -42 | 0.19 (0.36-0.09) | 24 | 0.35 (0.58-0.17) | 8 |
| Female | 0.21 (0.44-0.07) | -16 | 0.14 (0.29-0.05) | -40 | 0.21 (0.42-0.07) | 34 | 0.39 (0.77-0.15) | 64 |
| Male | 0.17 (0.36-0.06) | -22 | 0.09 (0.21-0.03) | -45 | 0.18 (0.44-0.06) | 15 | 0.31 (0.62-0.12) | -24 |
| Endocarditis |  |  |  |  |  |  |  |  |
| Both | 0.05 (0.1-0.02) | -51 | 0.04 (0.08-0.02) | -65 | 0.07 (0.15-0.03) | -27 | 0.24 (0.45-0.13) | -35 |
| Female | 0.04 (0.1-0.01) | -42 | 0.04 (0.09-0.01) | -59 | 0.07 (0.16-0.02) | -12 | 0.29 (0.59-0.13) | 4 |
| Male | 0.05 (0.14-0.01) | -56 | 0.04 (0.09-0.01) | -69 | 0.07 (0.18-0.02) | -37 | 0.19 (0.48-0.08) | -58 |
| Hypertensive heart disease |  |  |  |  |  |  |  |  |
| Both | ND | ND | ND | ND | ND | ND | 0.1 (0.16-0.05) | 22 |
| Female | ND | ND | ND | ND | ND | ND | 0.13 (0.26-0.06) | 97 |
| Male | ND | ND | ND | ND | ND | ND | 0.06 (0.12-0.03) | -30 |
| Ischemic heart disease |  |  |  |  |  |  |  |  |
| Both | ND | ND | ND | ND | ND | ND | 2.87 (4.26-1.77) | 33 |
| Female | ND | ND | ND | ND | ND | ND | 3.67 (6.12-1.97) | 61 |
| Male | ND | ND | ND | ND | ND | ND | 2.13 (3.67-1.09) | 4 |
| Non-rheumatic valvular heart disease |  |  |  |  |  |  |  |  |
| Both | ND | ND | ND | ND | ND | ND | 0.03 (0.08-0.01) | 65 |
| Female | ND | ND | ND | ND | ND | ND | 0.04 (0.12-0.01) | 105 |
| Male | ND | ND | ND | ND | ND | ND | 0.02 (0.06-0) | 14 |
| Pulmonary arterial hypertension |  |  |  |  |  |  |  |  |
| Both | 0.02 (0.06-0) | -46 | 0.01 (0.02-0) | -63 | 0.01 (0.03-0) | -25 | 0.03 (0.09-0.01) | -28 |
| Female | 0.02 (0.07-0) | -42 | 0.01 (0.03-0) | -61 | 0.02 (0.05-0) | -20 | 0.05 (0.14-0.01) | -7 |
| Male | 0.02 (0.06-0) | -48 | 0.01 (0.03-0) | -65 | 0.01 (0.03-0) | -30 | 0.02 (0.06-0) | -54 |
| Rheumatic heart disease |  |  |  |  |  |  |  |  |
| Both | 0.15 (0.28-0.06) | -80 | 0.29 (0.48-0.14) | -79 | 0.9 (1.43-0.52) | -56 | 1.45 (2.45-0.79) | -59 |
| Female | 0.14 (0.36-0.04) | -83 | 0.33 (0.74-0.1) | -82 | 1 (1.93-0.4) | -62 | 1.72 (3.51-0.62) | -55 |
| Male | 0.15 (0.33-0.05) | -77 | 0.25 (0.49-0.09) | -73 | 0.81 (1.48-0.36) | -47 | 1.2 (2.27-0.42) | -63 |
| Stroke |  |  |  |  |  |  |  |  |
| Both | 0.46 (0.74-0.27) | -31 | 0.41 (0.66-0.23) | -54 | 0.71 (1.15-0.41) | -6 | 2.86 (4.33-1.73) | -14 |
| Female | 0.5 (0.94-0.23) | -36 | 0.52 (0.94-0.23) | -56 | 0.82 (1.6-0.35) | -10 | 3.58 (6.33-1.94) | 6 |
| Male | 0.42 (0.77-0.19) | -25 | 0.3 (0.56-0.13) | -49 | 0.61 (1.14-0.29) | -1 | 2.18 (3.64-1.07) | -33 |
| Others |  |  |  |  |  |  |  |  |
| Both | 0.24 (0.42-0.12) | -4 | 0.19 (0.32-0.09) | -32 | 0.4 (0.71-0.2) | 39 | 0.19 (0.34-0.09) | 22 |
| Female | 0.23 (0.48-0.07) | 0 | 0.21 (0.44-0.07) | -30 | 0.42 (0.88-0.13) | 47 | 0.23 (0.46-0.08) | 72 |
| Male | 0.25 (0.51-0.1) | -7 | 0.16 (0.36-0.06) | -34 | 0.39 (0.77-0.15) | 31 | 0.15 (0.31-0.06) | -14 |

ND, no data; (Δ), prevalence shift from 1990 to 2021

Table S5. DALY rate and its shift from 1990 to 2023 of pediatric cardiovascular diseases among populations aged <28 days to 23 months

| Disease sub-category | < 28 days | | 1 – 5 months | | 6 – 11 months | | 12 – 23 months | |
| --- | --- | --- | --- | --- | --- | --- | --- | --- |
|  | DALY (95% UI) | Δ (%) | DALY (95% UI) | Δ (%) | DALY (95% UI) | Δ (%) | DALY (95% UI) | Δ (%) |
| Cardiovascular diseases |  |  |  |  |  |  |  |  |
| Both | 3567.01 (5171.52-2313.91) | -34 | 1650.64 (2528.28-976.9) | -31 | 418.14 (649.01-240.31) | -35 | 156.66 (226-98.05) | -41 |
| Female | 3881.69 (6244.15-2261.7) | -36 | 1757.14 (2973.9-912.3) | -30 | 477.63 (885.42-218.74) | -35 | 170.4 (290.66-92.13) | -52 |
| Male | 3269.41 (5218.33-1610.29) | -32 | 1549.79 (2727.5-740.58) | -32 | 361.77 (616.17-163.72) | -35 | 143.62 (228.3-74.93) | -20 |
| Congenital heart anomalies |  |  |  |  |  |  |  |  |
| Both | 120809.85 (179029.9-76113.69) | -13 | 15690.43 (22398.23-10166.42) | -23 | 4425.8 (6689.07-2779.83) | -21 | 971.43 (1427.26-624.15) | -21 |
| Female | 94707.7 (156908.45-44708.79) | -23 | 13227.8 (22318.33-6259.97) | -24 | 3969.84 (6672.39-1925.96) | -31 | 849.02 (1389.05-420.29) | -40 |
| Male | 145496.05 (247617.53-72002.88) | -6 | 18022.62 (29771.88-9692.12) | -22 | 4857.78 (8934.14-2639.59) | -11 | 1087.59 (1845.12-590.36) | 3 |
| Cardiomyopathy and myocarditis |  |  |  |  |  |  |  |  |
| Both | 398.72 (872.55-135.58) | -24 | 368.15 (708.37-142.14) | -23 | 139.83 (251.78-59.64) | -29 | 26.6 (49.41-11.35) | -21 |
| Female | 401.87 (895.5-108.72) | -16 | 355.59 (837.95-87.87) | -15 | 152.44 (350.03-42.41) | -25 | 30.04 (70.37-10.05) | -31 |
| Male | 395.75 (1299.9-96.82) | -30 | 380.04 (919.63-109.93) | -29 | 127.89 (255.93-45.77) | -34 | 23.34 (52.77-9.26) | -3 |
| Endocarditis |  |  |  |  |  |  |  |  |
| Both | 67.84 (158.2-26.87) | -50 | 73.02 (146.89-27.96) | -54 | 24.79 (48.1-9.63) | -58 | 8.08 (17.74-3.34) | -52 |
| Female | 53.16 (120.62-16.72) | -36 | 64.46 (149.02-16.03) | -43 | 29.2 (73.06-7.02) | -53 | 9.22 (21.5-3.12) | -55 |
| Male | 81.72 (211.66-24.65) | -56 | 81.13 (219.98-21.63) | -60 | 20.61 (47.9-6.11) | -64 | 6.99 (18.18-2.17) | -47 |
| Pulmonary arterial hypertension |  |  |  |  |  |  |  |  |
| Both | 261.55 (761.83-43.72) | -53 | 54.17 (152.44-11.4) | -48 | 18.87 (59.53-4.68) | -54 | 3.97 (11.62-1.09) | -50 |
| Female | 316.21 (979.05-47.54) | -51 | 58.27 (189.46-8.44) | -44 | 20.49 (74.96-2.9) | -53 | 4.71 (15.62-0.87) | -57 |
| Male | 209.86 (817.55-15.46) | -55 | 50.29 (177.39-6.58) | -52 | 17.33 (64.52-3.01) | -56 | 3.27 (12.21-0.62) | -36 |
| Rheumatic heart disease |  |  |  |  |  |  |  |  |
| Both | ND | ND | ND | ND | ND | ND | 14.19 (27.71-6.26) | -81 |
| Female | ND | ND | ND | ND | ND | ND | 16 (38.58-4.64) | -85 |
| Male | ND | ND | ND | ND | ND | ND | 12.48 (25.9-4.34) | -71 |
| Stroke |  |  |  |  |  |  |  |  |
| Both | 2529.97 (3987.94-1605.91) | -34 | 986.15 (1648.69-547.05) | -33 | 165.54 (305.59-87.93) | -37 | 57.34 (89.75-35.06) | -32 |
| Female | 2844.18 (4697.94-1436.24) | -38 | 1117.58 (2129.74-525.22) | -35 | 200.59 (413.7-77.56) | -41 | 64.93 (113.71-32.46) | -44 |
| Male | 2232.81 (3935.09-1099.99) | -29 | 861.67 (1670.84-401.22) | -30 | 132.33 (259.93-56.32) | -31 | 50.14 (92.11-25.48) | -6 |
| Others |  |  |  |  |  |  |  |  |
| Both | 308.93 (597.03-135.91) | -9 | 169.15 (317.41-71.37) | -6 | 69.11 (132.48-31.55) | -15 | 46.47 (76.14-23.4) | -6 |
| Female | 266.27 (710.66-80.91) | 1 | 161.24 (424.95-39.18) | 1 | 74.91 (179.61-21.45) | -12 | 45.49 (99.27-16.44) | -21 |
| Male | 349.27 (753.17-112.99) | -15 | 176.65 (398.29-55.34) | -11 | 63.61 (131.36-22.98) | -18 | 47.41 (85.85-19.87) | 16 |

ND, no data; (Δ), prevalence shift from 1990 to 2021.

Table S6. DALY rate and its shift from 1990 to 2021 of pediatric cardiovascular diseases among populations aged 2 to 19 years

| Disease sub-category | 2-4 years | | 5-9 years | | 10-14 years | | 15-19 years | |
| --- | --- | --- | --- | --- | --- | --- | --- | --- |
|  | DALY (95% UI) | Δ (%) | DALY (95% UI) | Δ (%) | DALY (95% UI) | Δ (%) | DALY (95% UI) | Δ (%) |
| Cardiovascular diseases |  |  |  |  |  |  |  |  |
| Both | 118.4 (166.98-80.33) | -41 | 130.42 (171.61-94.34) | -54 | 242.39 (316.6-176.59) | -26 | 676.14 (888.69-492.22) | -18 |
| Female | 124.04 (195.65-76.69) | -43 | 151.77 (216.87-98.19) | -57 | 265.18 (377.78-178.21) | -31 | 827.41 (1231.94-564.9) | -2 |
| Male | 113.02 (182.18-65.25) | -38 | 110 (160.88-73.09) | -48 | 220.85 (321.27-146.82) | -19 | 534.34 (786.33-336.69) | -33 |
| Congenital heart anomalies |  |  |  |  |  |  |  |  |
| Both | 376.87 (539.67-253.38) | -29 | 146.32 (210.35-97.04) | -27 | 144.8 (206.4-99.69) | -3 | 134.2 (191.36-89.53) | 4 |
| Female | 305 (480.82-169.58) | -45 | 124.34 (188.6-74.36) | -41 | 118.31 (178.68-73.17) | -18 | 120.17 (188.03-68.88) | -1 |
| Male | 445.3 (730.8-253.23) | -14 | 167.34 (277.18-92.82) | -12 | 169.85 (268.65-98.69) | 10 | 147.35 (233.31-87.6) | 8 |
| Aortic aneurysm |  |  |  |  |  |  |  |  |
| Both | ND | ND | ND | ND | ND | ND | 1.73 (2.88-0.97) | 63 |
| Female | ND | ND | ND | ND | ND | ND | 1.89 (3.52-0.93) | 88 |
| Male | ND | ND | ND | ND | ND | ND | 1.59 (3.58-0.64) | 41 |
| Cardiomyopathy and myocarditis |  |  |  |  |  |  |  |  |
| Both | 18.25 (31.56-9.12) | -15 | 11.18 (18.53-5.64) | -37 | 16.56 (29.35-8.28) | 26 | 27.19 (44.27-14.54) | 10 |
| Female | 19.68 (39.6-7.38) | -12 | 13.17 (25.74-5.46) | -37 | 17.66 (33.35-6.61) | 35 | 29.67 (57.46-13) | 64 |
| Male | 16.88 (33.48-7.36) | -17 | 9.28 (19.97-3.92) | -37 | 15.52 (35.16-6.37) | 18 | 24.86 (48.08-10.8) | -20 |
| Endocarditis |  |  |  |  |  |  |  |  |
| Both | 4.13 (8.92-1.87) | -50 | 3.28 (6.9-1.54) | -64 | 5.47 (11.78-2.53) | -27 | 17.4 (32.47-9.3) | -35 |
| Female | 3.8 (9.09-1.27) | -40 | 3.3 (7.43-1.14) | -57 | 5.33 (12.57-1.87) | -12 | 21.15 (43.22-9.53) | 4 |
| Male | 4.45 (11.98-1.47) | -55 | 3.26 (7.91-1.24) | -68 | 5.6 (13.84-1.95) | -37 | 13.89 (35.3-5.62) | -58 |
| Hypertensive heart disease |  |  |  |  |  |  |  |  |
| Both | ND | ND | ND | ND | ND | ND | 7.23 (12.02-3.93) | 21 |
| Female | ND | ND | ND | ND | ND | ND | 9.72 (18.91-4.69) | 92 |
| Male | ND | ND | ND | ND | ND | ND | 4.9 (8.78-2.25) | -29 |
| Ischemic heart disease |  |  |  |  |  |  |  |  |
| Both | ND | ND | ND | ND | ND | ND | 212.3 (312.35-132.18) | 33 |
| Female | ND | ND | ND | ND | ND | ND | 270.2 (446.4-146.03) | 60 |
| Male | ND | ND | ND | ND | ND | ND | 158.03 (268.96-82.93) | 5 |
| Non-rheumatic valvular heart disease |  |  |  |  |  |  |  |  |
| Both | ND | ND | ND | ND | ND | ND | 2.19 (5.59-0.73) | 64 |
| Female | ND | ND | ND | ND | ND | ND | 3.19 (8.55-0.84) | 104 |
| Male | ND | ND | ND | ND | ND | ND | 1.25 (4-0.35) | 13 |
| Pulmonary arterial hypertension |  |  |  |  |  |  |  |  |
| Both | 1.59 (4.91-0.45) | -45 | 0.76 (2.02-0.22) | -62 | 1.01 (2.71-0.32) | -24 | 2.58 (6.61-0.85) | -28 |
| Female | 1.51 (5.78-0.29) | -42 | 0.81 (2.55-0.18) | -60 | 1.24 (3.83-0.27) | -19 | 3.76 (10-0.91) | -7 |
| Male | 1.66 (5.11-0.34) | -48 | 0.71 (2.14-0.15) | -64 | 0.8 (2.18-0.17) | -29 | 1.48 (4.19-0.32) | -53 |
| Rheumatic heart disease |  |  |  |  |  |  |  |  |
| Both | 14.91 (26.6-7.01) | -78 | 29.41 (46.57-16.62) | -75 | 79.02 (119.65-48.92) | -53 | 121.48 (195.66-70.61) | -56 |
| Female | 14.53 (33.55-5.34) | -81 | 32.8 (66.16-14.1) | -79 | 86.18 (159.34-37.97) | -59 | 140.9 (274.47-57.14) | -52 |
| Male | 15.27 (31.03-5.66) | -74 | 26.16 (45.87-12.06) | -68 | 72.26 (124.01-37.31) | -44 | 103.27 (180.52-43.24) | -59 |
| Stroke |  |  |  |  |  |  |  |  |
| Both | 54.76 (79.54-37.19) | -27 | 61.85 (84.28-43.83) | -41 | 97.21 (129.95-69.55) | -10 | 265.64 (380.33-180.36) | -14 |
| Female | 60.93 (99.64-36.07) | -30 | 75.58 (111.63-47.64) | -44 | 111.58 (173.4-74.57) | -12 | 326.44 (524.44-212.41) | 1 |
| Male | 48.88 (79.87-29.27) | -23 | 48.72 (70.55-33.07) | -37 | 83.63 (126.75-52.95) | -6 | 208.65 (316.31-131.04) | -29 |
| Others |  |  |  |  |  |  |  |  |
| Both | 24.75 (40.41-13.33) | 1 | 23.95 (37.42-13.66) | -16 | 43.11 (68.89-26.1) | 45 | 18.39 (30.77-10.32) | 25 |
| Female | 23.59 (45.21-9.43) | 6 | 26.11 (44.49-13.3) | -14 | 43.19 (77.77-19.43) | 55 | 20.49 (37.76-9.04) | 71 |
| Male | 25.87 (49.21-11.91) | -2 | 21.88 (38.24-11.69) | -18 | 43.05 (69.39-23.32) | 37 | 16.42 (28.39-8.9) | -6 |

ND, no data; (Δ), prevalence shift from 1990 to 2021

Table S7. GAM-based estimates and inflection years for CHA and non-congenital CVDs by age group and sex.

| Age and sex | CHA | | | | | | Non-congenital CVD | | | | | |
| --- | --- | --- | --- | --- | --- | --- | --- | --- | --- | --- | --- | --- |
|  | EDF | F | p-value | Adj. R^2^ | Deviation explained | Inflection year | EDF | F | p-value | Adj. R^2^ | Deviation explained | Inflection year |
| <28 days |  |  |  |  |  |  |  |  |  |  |  |  |
| Both | 2.364 | 14.62 | <0.001 | 0.292 | 30.8 | 2018 | 1.843 | 16.65 | <0.001 | 0.268 | 28.2 | NA |
| Female | 2.863 | 37.06 | <0.001 | 0.565 | 57.7 | 2018 | 1.947 | 23.29 | <0.001 | 0.354 | 36.6 | NA |
| Male | 2.036 | 8.07 | <0.001 | 0.16 | 17.7 | 2020 | 1.734 | 12.36 | <0.001 | 0.202 | 21.6 | NA |
| 1-5 months |  |  |  |  |  |  |  |  |  |  |  |  |
| Both | 6.582 | 174.03 | <0.001 | 0.93 | 93.5 | 1994 | 2.255 | 15.98 | <0.001 | 0.3 | 31.5 | NA |
| Female | 5.336 | 82.11 | <0.001 | 0.84 | 84.8 | 1994 | 4.563 | 53.39 | <0.001 | 0.747 | 75.8 | 1994 |
| Male | 6.335 | 141.7 | <0.001 | 0.913 | 91.8 | 1994 | 1.569 | 6.78 | 0.004 | 0.1 | 11.4 | NA |
| 6-11 months |  |  |  |  |  |  |  |  |  |  |  |  |
| Both | 3.614 | 46.72 | <0.001 | 0.674 | 68.5 | 2017 | 1.002 | 14.62 | <0.001 | 0.119 | 12.8 | NA |
| Female | 1.938 | 43.64 | <0.001 | 0.508 | 51.7 | NA | 1.193 | 27.41 | <0.001 | 0.256 | 26.5 | NA |
| Male | 5.112 | 63.63 | <0.001 | 0.796 | 80.7 | 1993 | 1.002 | 7.93 | 0.006 | 0.064 | 7.4 | NA |
| 12-23 months |  |  |  |  |  |  |  |  |  |  |  |  |
| Both | 1.32 | 31.66 | <0.001 | 0.312 | 32.1 | NA | 1.001 | 22.49 | <0.001 | 0.176 | 18.4 | NA |
| Female | 1.011 | 53.37 | <0.001 | 0.346 | 35.2 | NA | 1.001 | 34.28 | <0.001 | 0.248 | 25.6 | NA |
| Male | 1.572 | 16.35 | <0.001 | 0.225 | 23.7 | NA | 1.001 | 12.27 | 1.00E-03 | 0.1 | 10.9 | NA |
| 2-4 years |  |  |  |  |  |  |  |  |  |  |  |  |
| Both | 1.513 | 51.51 | <0.001 | 0.473 | 48.1 | NA | 1.001 | 40.57 | <0.001 | 0.282 | 28.9 | NA |
| Female | 1.001 | 70.12 | <0.001 | 0.407 | 41.2 | NA | 1.001 | 44.68 | <0.001 | 0.302 | 30.9 | NA |
| Male | 2.054 | 36.13 | <0.001 | 0.475 | 48.6 | NA | 1.001 | 35.22 | <0.001 | 0.253 | 26.1 | NA |
| 5-9 years |  |  |  |  |  |  |  |  |  |  |  |  |
| Both | 1.001 | 71.23 | <0.001 | 0.411 | 41.6 | NA | 2.297 | 164.28 | <0.001 | 0.823 | 82.7 | NA |
| Female | 1.012 | 66.76 | <0.001 | 0.399 | 40.5 | NA | 3.41 | 310.12 | <0.001 | 0.928 | 93.1 | NA |
| Male | 1 | 64.24 | <0.001 | 0.385 | 39.1 | NA | 1 | 114.27 | <0.001 | 0.529 | 53.3 | NA |
| 10-14 years |  |  |  |  |  |  |  |  |  |  |  |  |
| Both | 1 | 32.12 | <0.001 | 0.236 | 24.3 | NA | 1.657 | 110.81 | <0.001 | 0.692 | 69.7 | NA |
| Female | 1.001 | 18.64 | <0.001 | 0.149 | 15.7 | NA | 3.418 | 39.38 | <0.001 | 0.622 | 63.5 | NA |
| Male | 3.127 | 14.58 | <0.001 | 0.359 | 37.8 | 1996 | 3.68 | 51.39 | <0.001 | 0.698 | 70.9 | 2000 |
| 15-19 years |  |  |  |  |  |  |  |  |  |  |  |  |
| Both | 1 | 21.04 | <0.001 | 0.166 | 17.4 | NA | 2.299 | 5.62 | 0.002 | 0.127 | 14.6 | 2002 |
| Female | 1 | 28.9 | <0.001 | 0.217 | 22.4 | NA | 1.879 | 1 | 0.477 | 0.011 | 2.9 | 2007 |
| Male | 1 | 12.33 | <0.001 | 0.101 | 11 | NA | 2.762 | 23.58 | <0.001 | 0.442 | 45.8 | 1998 |
